# Disparate privacy risks from medical AI

**DOI:** 10.1038/s41586-026-10688-0

**Published:** 2026-06-24

**Authors:** Moritz A. Knolle, Martin J. Menten, Friederike Jungmann, Felix Meissen, Ben Glocker, Daniel Rueckert, Georgios Kaissis

**Affiliations:** 1 Chair for AI in Healthcare and Medicine, Technical University of Munich (TUM) and TUM University Hospital, Munich, Germany; 2 Munich Center for Machine Learning, Munich, Germany; 3 Department of Computing, Imperial College London, London, UK; 4 Institute for Diagnostic and Interventional Radiology, TUM University Hospital, Technical University of Munich, Munich, Germany; 5 Hasso Plattner Institute for Digital Engineering, University of Potsdam, Potsdam, Germany

**Keywords:** Medical imaging, Computational science, Information technology, Risk factors

## Abstract

Medical artificial intelligence (AI) models hold the promise to improve global access to high-quality diagnostics^1^. However, the training data underlying these models often contain sensitive patient information that may be exposed through privacy attacks^2–7^. Previous research has primarily quantified the success of these attacks in aggregate, across all records in a dataset. Thus, the privacy risk faced by individual patients, who often contribute multiple similar records to a training dataset, is poorly understood. Here we present one of the first patient-level privacy audits of AI models for medical diagnostic applications. We focus on membership inference attacks^2–4^ (MIAs), which seek to determine whether the data of a given individual were used to train a model. Across a diverse range of medical datasets, we show that MIAs can achieve near-perfect success rates for individual patients, even when the aggregate performance does not substantially deviate from random guessing. We further find that the number of patients with high attack success increases substantially with model capacity, and that underrepresented groups—stratified by disease status, self-reported race, insurance, sex or imaging protocol—face disproportionately high attack success. Together, our findings show that aggregate privacy metrics can severely underestimate individual privacy risk. Whether the disparate risk profiles we observe extend to attacks beyond MIAs remains an open question, motivating the further development of risk assessment and mitigation techniques that cater to all data-contributing patients.

## Abstract (web summary)

AI models for medical diagnostics are vulnerable to membership inference attacks.

## Main

Medical artificial intelligence (AI) has immense potential to improve health outcomes, particularly in regions in which specialized medical expertise is scarce^1^. At the same time, AI also poses new challenges and risks, including security vulnerabilities that arise when models are deployed. Untrusted users with access to an AI model may, by merely observing its predictions, steal its parameters^8,9^ or perform privacy attacks^2–7^, which can extract sensitive details about the data used for model training.

Privacy attacks against an AI model can enable detailed inferences about the individuals who contributed to its training data. For example, a membership inference attack (MIA)^2^ attempts to determine whether the data of a specific patient were included in the training dataset of a model. The extent to which this constitutes a privacy violation is nuanced and depends on factors such as the underlying training population and the deployment context of the model. Although inferring membership for a model trained on a general population may be benign, doing so for a model trained on a narrow, disease- or centre-specific cohort acts as a direct proxy for sensitive medical information. For example, a successful MIA against the model in ref. ^10^, which predicts anti-cancer immunotherapy efficacy from routine blood test data, reveals that an individual has cancer.

The accelerating deployment of medical AI models trained on sensitive patient data^11^ calls for rigorous privacy risk assessments. However, previous studies primarily quantified the success rate of MIAs, in aggregate, across all records in a training dataset. This implicitly averages risk across records, thereby obscuring important information on record- and patient-level attack success. Consequently, the risk that an individual faces by contributing their personal data (often multiple records) to an AI training dataset is poorly understood. Given that medical data are a key target for cybercriminals^12,13^, and pseudonymization alone is increasingly recognized as insufficient to prevent the re-identification of individuals in large, high-dimensional datasets^14–16^, there is a need to improve our understanding of the threat that AI privacy attacks pose to individual patients.

Here we show that deploying medical AI models without protective measures can pose substantial privacy risks to individual data-contributing patients. These risks are particularly acute when membership in a training population itself reveals sensitive medical information. Our privacy audit of AI models trained to perform standard diagnostic (supervised classification) tasks quantifies state-of-the-art MIA success^3,4^ at the resolution of individual data contributors. Using seven large datasets comprising real-world clinical data, including various types of medical images, electrocardiograms and electronic health records, we demonstrate that the success of a MIA is unequally distributed among data-contributing patients. We show that this disparity exists at two levels: (1) the individual patient level, at which some patients experience near-perfect attack success, whereas others remain essentially unaffected; and (2) the group level, at which patient groups underrepresented in a training dataset are often overrepresented among records most vulnerable to MIAs.

Together, our results indicate that privacy attacks against AI models may be much more effective at compromising the privacy of individual data contributors than previously thought. This suggests that current AI privacy risk reporting practices may underestimate individual-level risk and thus motivates the integration of mathematically verifiable risk mitigation strategies such as differential privacy (DP) into medical AI model development workflows.

### Attacking AI by simple hypothesis tests

**Fig. 1:**
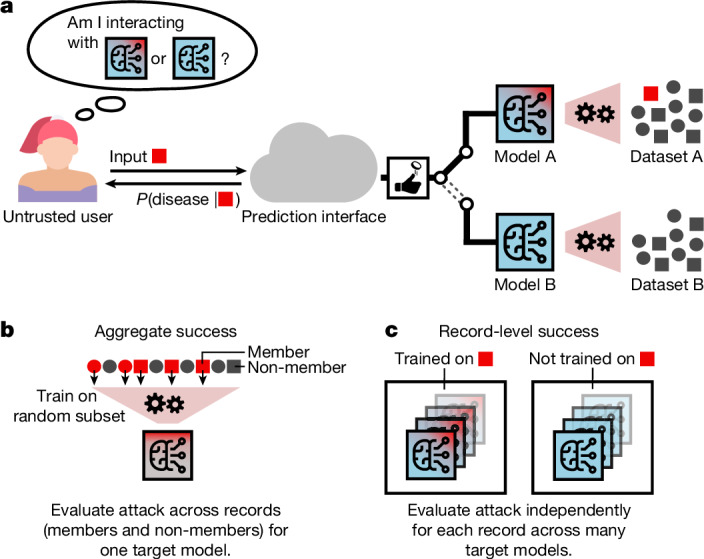
MIA and evaluation strategies. **a**, Schematic of a MIA, in which an untrusted user, only by observing the predictions of a model, aims to infer whether a specific target record was part of the training dataset. The attack is considered successful if the untrusted user can reliably distinguish between model A and model B, which are identical except for the inclusion and exclusion of the target record in the respective training dataset. **b**,**c**, Attack success can be measured either, in aggregate, across all records in the dataset (**b**) or, more granularly, for each record individually across many target models (**c**).

A popular deployment strategy for AI models gives users access to a model through a prediction interface, which, for a given input (for example, the chest radiograph of a patient), returns a corresponding prediction (for example, a 78% chance of pneumonia). This black-box access to a model can be exploited by an untrusted user to conduct a MIA that shows the membership status of a target record, that is, whether the target record was a member of the training dataset of a model or not (Fig. 1a). To infer membership status, MIAs typically make use of the fact that AI models are often slightly more confident about their predictions on training than on non-training data.

Likelihood-ratio MIAs (LR-MIAs)^3,4^, the current state-of-the-art in MIAs, frame membership inference as a simple vs. simple hypothesis testing problem on the prediction confidence provided by the target model. In essence, LR-MIAs compare the likelihood of the predicted confidence of the target model for the target record under the null (the target record was not a member) and the alternative hypothesis (the target record was a member). Here, the parameters of the distributions under the two hypotheses are specified by parametric fitting of sample confidence values obtained from reference models. Reference models are models assumed to be trained by the attacker and are ideally, but not necessarily, of similar architecture as the target model and trained on data similar to the training dataset of the target model.

Note that objectively larger threats are posed by privacy attacks with stronger assumptions on a potential attacker, such as access to model parameters^17^, access to parameter updates during model training^18^ or, furthermore, the ability to modify the model architecture^19,20^. However, we do not consider them in this study as their strong assumptions are not realistic for careful, practical deployment scenarios. By contrast, the type of attack we consider here requires querying the target model only once (to obtain a prediction for the target record) and may thus be executed by any attacker posing as a real user of an AI system. Notably, as the attacks we study are executed against fully trained models, data-governance-preserving techniques such as federated/swarm-learning^21^ provide no protection.

### From aggregate to patient-level risk

MIA performance is evaluated through a receiver operating characteristic (ROC) analysis^22^ on numerous repetitions of the MIA game scenario, in which an untrusted user is challenged to guess the membership status of a given record (Fig. 1a). In practice, owing to the computational cost of training AI models, attack success is typically evaluated using a single target model. More specifically, a target model is trained on a random subset of the training dataset, and subsequently, an ROC analysis is performed on the aggregated membership predictions for all records in the dataset (Fig. 1b). Although practical, this approach has a key shortcoming: it provides no indication of the performance of the attack for individual records or patients.

To address this issue, we propose a simple technique for estimating record-, and by extension, patient-level vulnerability to LR-MIAs (Fig. 1c). In brief, using a large set of target models (*N* = 200) trained on random patient subsets, we estimate, for each training record, sampling distributions of the confidence of the target model under the null and alternative hypotheses in LR-MIAs. In other words, we estimate empirical distributions of confidence values as provided by target models, partitioned into models trained and not trained on the target record. Because these distributions are assumed to take Gaussian form in LR-MIAs^3,4^, record-level attack success, as measured by the area under the ROC curve (AUC), can be calculated in closed form (Methods). A high AUC score, close to the maximum value of 1.0, suggests high privacy risk: a MIA for this record could achieve high sensitivity with little to no false positives. Notably, the record-level MIA AUC also offers a probabilistic interpretation^22^: the record-level MIA AUC is the probability that a confidence score from a target model trained on the target record is larger than a score from a target model not trained on the target record.

**Extended Data Fig. 1:**
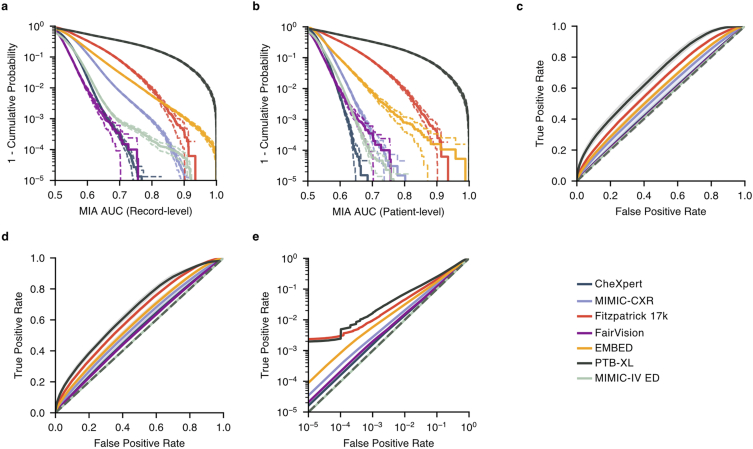
Additional MIA success data. **a**, eSF plots of raw record-level MIA AUC scores computed using *N* = 200 target models. **b**, eSF plots of mean patient-level MIA AUC scores computed using *N* = 200 target models. **c**–**e**, ROC analysis of aggregate MIA success (*R* = 190 reference models, online) for LiRA on a linear scale (**c**) and RMIA on linear (**d**) and log-log scale (**e**). ROC curve plots show mean and standard deviation (vertical average) aggregate attack success for *N* = 10 target models; on the log-log scale, the standard deviation was omitted to improve visibility. Dashed grey lines in ROC curve plots indicate random-guessing performance, dashed lines in eSF plots indicate 95% Greenwood CI.

Correctly determining the membership status for one of the records contributed by an individual patient reveals the membership status of the patient. Thus, we compute patient-level scores by taking the maximum across all record-level scores for a given patient. The raw record-level scores and the average patient-level scores can be found in Extended Data Fig. 1.

**Extended Data Fig. 2:**
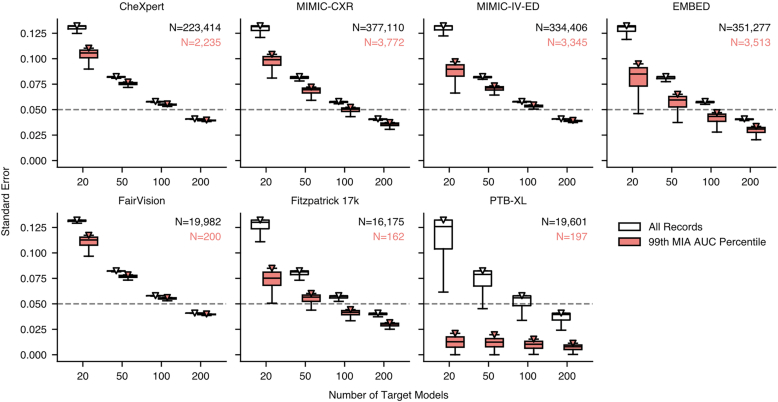
Ablation study on the number of target models used to estimate record-level MIA AUC scores. Panels show box plots of the standard error values for record-level MIA AUC scores in a given dataset when varying the number of target models used for record-level MIA AUC estimation. White box plots show data for all records in a given dataset, red box plots show data for the 99th record-level MIA AUC percentile. In box plots, the centre line indicates the median, box bounds represent 25th and 75th percentiles, whiskers extend to 1.5 times the interquartile range from the box bounds, and triangles indicate largest outlier value.

Notably, our technique for measuring record-level attack success reduces to estimating the bi-normal AUC from sample statistics and thus has desirable statistical properties. Its standard error at the record level can be computed in closed form^23^ (Methods). As expected, using a total of *N* = 200 target models evenly split between null and alternative hypotheses for each record, the standard error of the record-level MIA AUC is small across all records in the investigated datasets (Extended Data Fig. 2).

### Attacking open-source models

Recent advances in attack design have made LR-MIAs much more practical. To illustrate the practical feasibility of conducting MIAs, we demonstrate attacks against two chest radiograph models from the TorchXrayVision^24^ library. We used the Robust Membership Inference Attack^4^ (RMIA), an improved LR-MIA that requires only one or two reference models, compared with more than 100 for the Likelihood Ratio Attack^3^ (LiRA). RMIA achieves this efficiency gain by effectively using reference data (data similar to the target record) alongside the target record to query the target model. Crucially, the attack does not require knowledge of the membership status of the reference data.

We simulated a realistic attack setting in which an attacker lacks access to the training dataset of the target model to train reference models and is further constrained by computational resources. Specifically, we used only a single pre-trained PadChest^25^ model as a reference model to perform attacks against the CheXpert^26^ and MIMIC-CXR^27^ models of the library. In this setting, also known as an offline attack, an attacker incurs no computational cost in training the reference model. Instead, they simply need to obtain predictions from the reference model for both the target record and the reference data. This can be done efficiently on commodity hardware without a graphics processing unit (GPU). To conduct the attack, we queried the target model once to collect confidence values for all target records. Using this collection, we then computed RMIA test statistics for each target record by randomly selecting, independent of membership status, *N* = 500 confidence values from the other targets in this collection as reference data. This strategy would effectively conceal the additional reference data queries to the target model in a real attack.

**Fig. 2:**
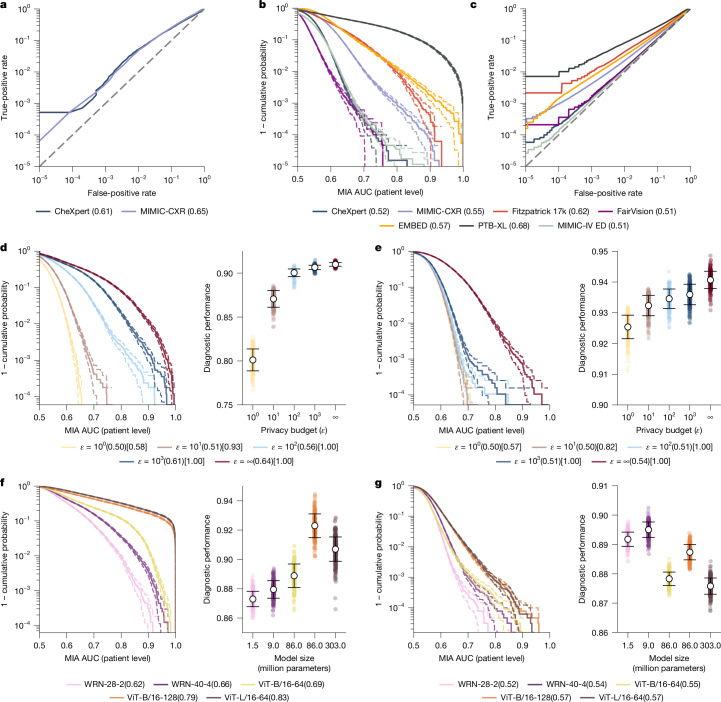
MIAs pose substantial privacy risks to individual data-contributing patients. **a**, Aggregate MIA success for a realistic attack against open-source CheXpert and MIMIC-CXR models (RMIA offline, *R* = 1 reference model pre-trained on PadChest). **b**, eSF analysis of patient-level MIA AUC scores computed using *N* = 200 target models for each dataset (residual networks, about 1.5 million parameters each). Patient-level scores are computed as the maximum record-level score for a given patient. **c**, ROC analysis of aggregate attack success (LiRA online, vertical-average mean for *N* = 10 target models, *R* = 190 reference models). **d**,**e**, eSF plots of patient-level MIA AUC scores alongside diagnostic performance (macro-average AUC) on unseen test data for *N* = 200 target models with varying levels of record-level (*ε*, *δ*)-DP privacy protection: PTB-XL (**d**) and EMBED (**e**). *δ* was kept constant at 1/*D*, where *D* is the dataset size. **f**,**g**, eSF plots of patient-level MIA AUC scores alongside diagnostic performance (macro-average AUC) on unseen test data for *N* = 200 target models of increasing model capacity: Fitzpatrick-17k (**f**) and CheXpert (**g**). Error bars indicate s.d., round brackets indicate aggregate attack AUC; square brackets indicate AUC upper bound implied by record-level DP accounting; dashed grey lines in ROC curve plots indicate random-guessing performance; and dashed lines in eSF plots indicate 95% Greenwood CI.

We evaluated attack success on a combined dataset of records from CheXpert and MIMIC-CXR (*N* = 25, 000 each), which were, respectively, labelled as members and non-members for the CheXpert model (v.v. for the MIMIC-CXR model). In this setting, RMIA achieved substantial aggregate success with respective AUC scores of 0.61 and 0.65 (Fig. 2a). Note that owing to the distribution shift between members and non-members, results from this evaluation setting are not directly comparable to the standard evaluation protocol in which members and non-members are sampled at random from the training dataset. Notably, however, such a distribution shift is expected in a real attack, and this setting is thus of high interest.

### Near-perfect success for some patients

After demonstrating realistic attacks against two open-source models, we next investigated how effectively MIAs can compromise the privacy of individual patients. To this end, we measured patient-level MIA success across a diverse range of medical datasets using, for each, a large set of target models. Notably, we used state-of-the-art model training techniques (for example, data augmentation, weight decay and learning rate schedules) and furthermore, took explicit countermeasures to prevent overfitting, which is known to exacerbate privacy risks^2,28^. As a result, the investigated target models, despite being trained on roughly half of the available data each, provide high diagnostic performance within a few percentage points of published baselines (Methods).

Across all investigated datasets and models, we identified a small subset of patients who are highly vulnerable to LR-MIAs. This is indicated by empirical survival functions (eSF) of patient-level MIA AUC scores, which, for a given score, show the proportion of patients with this score or higher (Fig. 2b). By contrast, ROC curves of aggregate attack success and their corresponding AUC scores do not deviate substantially from the random-guessing baseline, thus incorrectly indicating a low attack vulnerability (Fig. 2c and Extended Data Fig. 1c–e). This suggests that average-case metrics of attack success, as used in the standard evaluation protocol, are unsuitable measures of privacy risk. They do not accurately reflect that some records or patients may be highly vulnerable, whereas the vast majority are not.

**Extended Data Fig. 3:**
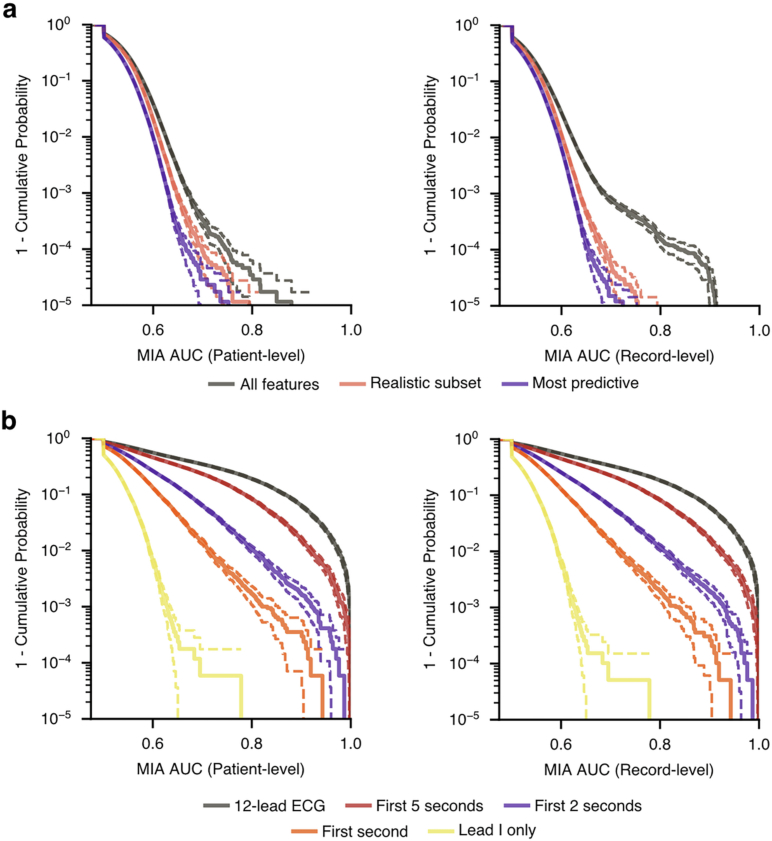
MIAs remain highly successful with partial target record access. **a**, Patient- and record-level MIA AUC scores for MIMIC-IV-ED. We simulate an attacker with access to: all clinical features (*F* = 64), a realistic subset of features related only to a patient’s current stay (*F* = 20) and the ten most predictive features for hospitalisation (*F* = 10). **b**, Patient- and record-level MIA AUC scores for PTB-XL. We simulate an attacker with access to: the full twelve-lead ECG signal, Lead I only, and a range of temporal snippets, each 1 to 5 seconds long. Dashed lines indicate 95% Greenwood confidence intervals; record-level MIA AUC scores were computed using *N* = 200 target models for each experimental setting.

For the two non-imaging datasets, MIMIC-IV-ED^29^ (electronic health records) and PTB-XL^30^ (electrocardiograms), we simulated attack settings in which an attacker only has partial access to the target record (Extended Data Fig. 3). Although MIA success generally decreases under partial data access, a subset of patients retain high AUC scores, even in settings in which the attacker has access to only basic clinical information—such as a patients’ age, sex, chief complaints and vital signs (MIMIC-IV-ED), or only the lead I signal from a 12-lead electrocardiogram (PTB-XL).

We verified how resolvable the discovered vulnerabilities are by training models with different levels of record-level (*ε*, *δ*)-DP protection (Fig. 2d,e). As expected, we find that patient-level MIA risk decreases with stronger levels of privacy protection (smaller *ε* values). Moreover, in most scenarios, we observe no violation of the record-level DP guarantee (indicated by the square brackets in the panel legend), although many patients contributed multiple records. Violations are observed only for a subset of patients under strong privacy protection (*ε* = 1), in which some patients have MIA AUC scores exceeding the upper bound on the MIA AUC implied by the record-level DP guarantee. This behaviour is expected and could be mitigated by implementing patient-level DP accounting.

### Larger models, greater risks

Many of the recent AI success stories have been driven not by methodological advances but by scaling up model and dataset sizes^31^. In light of this scaling trend, we next investigated the impact of model capacity on MIA success. For Fitzpatrick 17k^32^ and CheXpert, we trained models with increasing capacity, including wide residual networks^33^ (WRN-28-2 and WRN-40-4) and vision transformers^34^ (ViT-B/16 and ViT-L/16). Where computationally feasible, vision transformers were trained on images of different sizes: 64 × 64 and 128 × 128 pixels; this is indicated by a trailing number behind the model name (for example, ViT-B/16-64 and ViT-B/16-128).

We find that MIA success (both at the aggregate and patient levels) increases with model capacity. We observe that the relative share of patients highly vulnerable to MIAs increases greatly for larger models, often by an order of magnitude (Fig. 2f,g). For the dermatology dataset (Fitzpatrick 17k), increasing model capacity yields large gains in diagnostic performance with a pronounced increase between WRN-40-4 and ViT-B/16-128, which was pre-trained on a large dataset of more than 14 million natural images. However, simultaneously, the number of patients with near-perfect attack success (AUC score of 0.95 or higher) increases substantially: 0 (WRN-28-2), 1 out of 10,000 (WRN-40-4), 1 out of 1,000 (ViT-B/16-64) and 1 out of 10 (ViT-B/16-128). We observe a similar trend in the much larger dataset CheXpert, although attack success is generally lower. Notably, for CheXpert, vision transformer models do not achieve diagnostic performance competitive with WRN-based models. This is probably because of the diminished utility of natural-image pre-training for medical greyscale images^35^.

### Attack success varies by subgroup

Motivated by recent findings^36,37^, which revealed that the diagnostic performance of AI models can differ across patient subgroups, we investigated whether differences in privacy risk exist between subgroups. To this end, we focused our analysis on the most vulnerable records (99th MIA AUC percentile) and compared how frequently a subgroup appears in this extreme-risk tail compared with the overall dataset. We did not consider differences in aggregate attack success, as we previously identified this metric as an unsuitable measure of privacy risk.

**Fig. 3:**
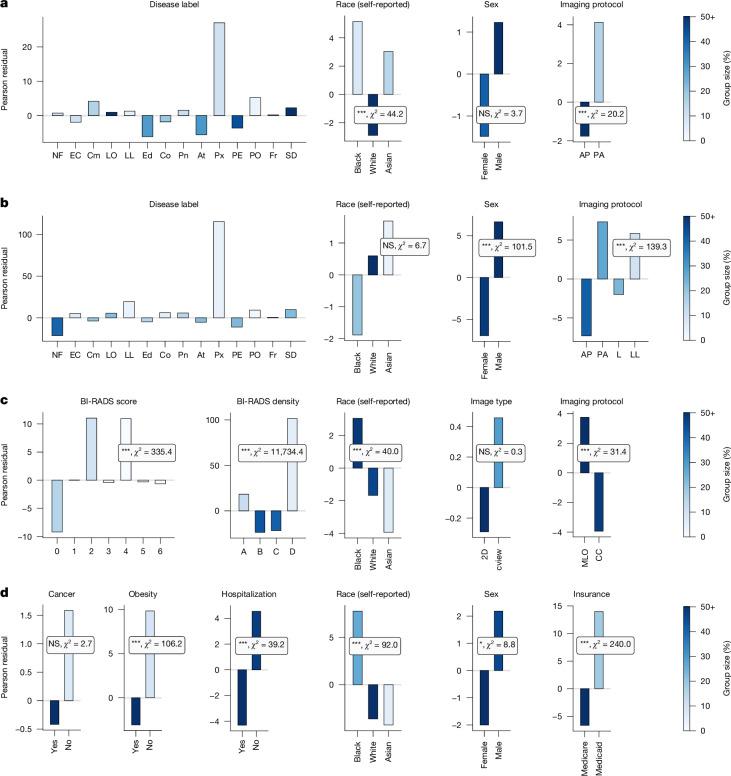
Significant differences in extreme MIA risk between patient subgroups. **a–d**, The panel rows show two-sided *χ*^2^-test results for subgroup counts among the 99th record-level MIA AUC percentile for CheXpert (**a**), MIMIC-CXR (**b**), EMBED (**c**) and MIMIC-IV-ED (**d**). Pearson residuals measure the contribution of a group to the test statistic. A large, positive value indicates that more records were observed for this group than expected. A negative value indicates the opposite. The column titles show stratification variables; bars are coloured according to the relative share of records of a group in the training dataset. *P ≤ 0.05, **P ≤ 0.01, ***P ≤ 0.001 and NS, not significant. Multiple comparison correction was applied row-wise using the Bonferroni method; statistical significance could not be tested for CheXpert and MIMIC-CXR disease label groups as the categorization is not mutually exclusive; race subgroup comparisons in EMBED are likely confounded by breast density differences. Left to right, CheXpert/MIMIC-CXR disease labels refer to no finding (NF), enlarged cardiomediastinum (EC), cardiomegaly (Cm), lung opacity (LO), lung lesion (LL), oedema (Ed), consolidation (Co), pneumonia (Pn), atelectasis (At), pneumothorax (Px), pleural effusion (PE), pleural other (PO), fracture (Fr) and support devices (SD). Imaging protocol abbreviations refer to anteroposterior (AP), posteroanterior (PA), lateral (L), lateral left (LL), mediolateral oblique (MLO) and craniocaudal (CC). BI-RADS indicates breast cancer assessment: incomplete (BI-RADS-0) to biopsy-proven malignancy (BI-RADS-6) and breast density: mostly fatty (BI-RADS-A) to extremely dense (BI-RADS-D). Left to right, adjusted *P* values are as follows. CheXpert: 8.1 × 10^−9^, 9.7 × 10^−2^, 2.1 × 10^−5^; MIMIC-CXR: 0.1, 2.2 × 10^−23^, 1.6 × 10^−29^; EMBED: 1.0 × 10^−68^, <1.0 × 10^−100^, 1.0 × 10^−8^, 1.0, 2.7 × 10^−7^. MIMIC-IV-ED: 0.61, 4.1 × 10^−24^, 2.3 × 10^−9^, 6.3 × 10^−20^, 1.8 × 10^−2^, 2.3 × 10^−53^.

**Extended Data Fig. 4:**
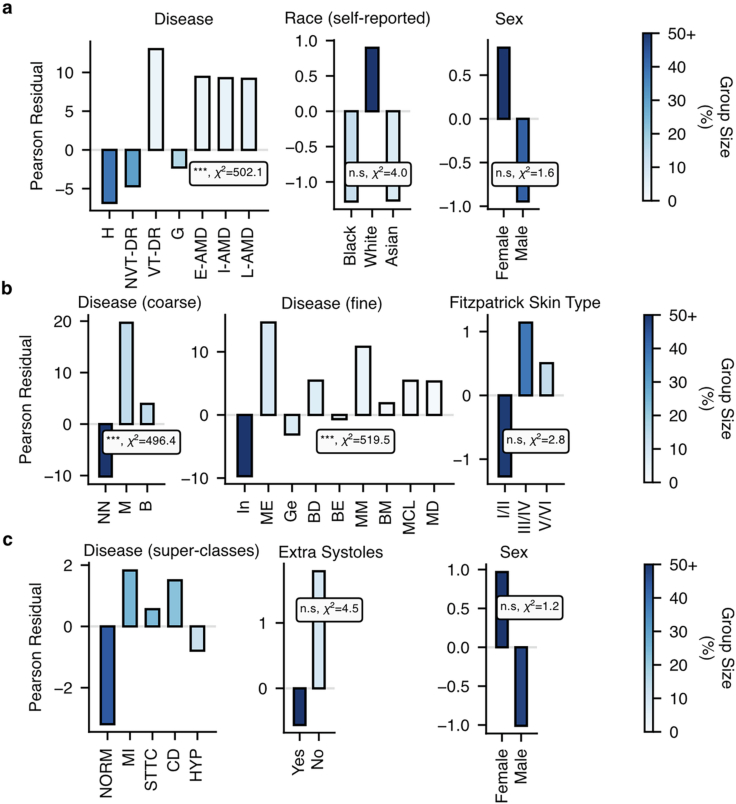
Subgroup differences in extreme MIA risk for FairVision, Fitzpatrick17k and PTB-XL. Panel rows show two-sided *χ*^2^-test results and Pearson residuals for subgroup counts in the set of highly vulnerable records (99th record-level MIA AUC percentile) for FairVision (**a**), Fitzpatrick 17k (**b**) and PTB-XL (**c**). Column titles show stratification variables; bars are coloured according to group size in the training dataset; *** and n.s. denote *p* ≤ 0.001 and not significant, respectively; multiple comparison correction was performed row-wise using the Bonferroni method. From left to right, the FairVision disease abbreviations refer to healthy, non-vision-threatening diabetic retinopathy; vision-threatening diabetic retinopathy; glaucoma; early age-related macular degeneration (AMD); intermediate AMD; and late AMD. From left to right, the Fitzpatrick 17k disease abbreviations refer to non-neoplastic, malignant, benign, inflammatory, malignant epidermal, genodermatoses, benign dermal, benign epidermal, malignant melanoma, benign melanocyte, malignant cutaneous lymphoma, and malignant dermal. Fitzpatrick skin type is a clinical categorisation of human skin colour that describes sensitivity to sun exposure, ranging from sensitive, always burns, never tans (Type I) to sun-insensitive, never burns, deeply pigmented (Type VI). From left to right adjusted *p* values read. Fairvision: < 1 × 10^−100^, 0.53, 0.85. Fitzpatrick 17k: < 1 × 10^−100^, < 1 × 10^−100^, 0.74. PTB-XL: 0.67, 0.53.

We find that extreme MIA risk is unequally distributed across patient subgroups when stratifying by disease status, self-reported race, sex, imaging protocol or health insurance. More precisely, for most comparisons, we observe significant differences in subgroup composition between the most vulnerable records and the overall dataset (Fig. 3 and Extended Data Fig. 4). For example, in MIMIC-IV-ED, records from Black patients, patients with Medicaid insurance or patients diagnosed with cancer were observed more frequently than expected among the most vulnerable records (+31%, +126%, and +18% relative change to the overall dataset, respectively). Raw data on the composition of the extreme MIA risk tails as well as the overall datasets are provided in Supplementary Tables 3–16. To find factors that could explain the observed differences, we performed a post hoc test analysis and computed Pearson residuals for all subgroup comparisons (Fig. 3 and Extended Data Fig. 4).

We primarily observe large, positive Pearson residuals for underrepresented groups in the datasets, suggesting that relative group size influences MIA risk. Consider, for example, EMBED^38^, a mammography dataset comprising mostly negative findings, that is, unremarkable mammograms of healthy breasts with no indication of a tumour. Models for this dataset are trained to predict breast density, and thus never have direct access to tumour findings. Despite this, benign tumour findings (BI-RADS-2) and tumour findings suspicious of malignancy (BI-RADS-4) account for a disproportionately large share of the most vulnerable records (+60% and +1,179% relative change to the overall dataset, respectively). Similarly, otherwise relatively uncommon images of almost entirely fatty (BI-RADS-A) or extremely dense (BI-RADS-D) breasts also occur disproportionately frequently (+90% and +755%, respectively).

**Extended Data Fig. 5:**
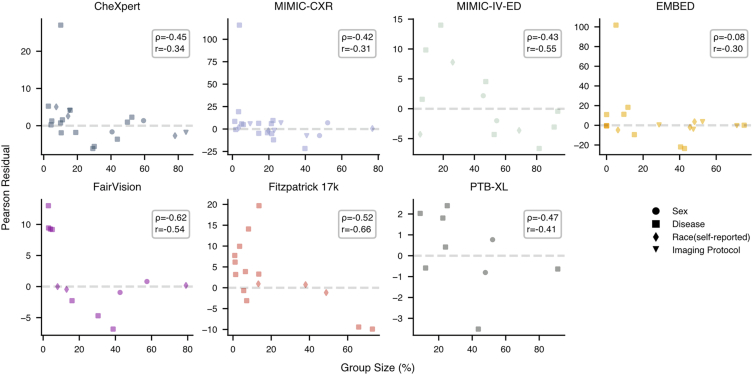
Underrepresented groups experience disproportionally high MIA risk. Scatter plot of Pearson residuals against group size (relative number of records contributed) for all subgroup comparisons. Across the investigated datasets, group size is weakly/moderately inversely correlated with Pearson residual as indicated by Spearman’s rank correlation coefficient *ρ* and Pearson’s correlation coefficient r.

To further investigate the relationship between group size and MIA risk, we conducted a meta-analysis of all computed Pearson residuals (Extended Data Fig. 5). Confirming previous observations, we find that large positive Pearson residuals occur mostly for small groups (those that contribute less than 20% of the records of a dataset). Moreover, we observe a weak to moderate negative correlation between group size and Pearson residuals. This suggests that the observed differences in MIA risk may, at least in part, be driven by group-size differences in the training data.

### Discussion

We present data from the first patient-level privacy audit of medical AI models. Our findings confirm early observations of MIA risk heterogeneity^39–42^ and, at the same time, substantially advance previous AI privacy auditing efforts along three key dimensions. First, our work marks a shift towards patient-level risk assessment, which is crucial for real-world clinical datasets, in which individuals often contribute multiple, similar records. Second, we demonstrate that aggregate success rates, as used in the standard evaluation protocol and previous subgroup analyses^41,42^, underestimate true privacy risks. Third, we confirm that MIA vulnerabilities previously observed on low-dimensional benchmark datasets^2–4,39–42^ are present, and arguably more critical, in large representative clinical datasets. Below, we briefly discuss our findings and their implications.

The fact that MIAs can achieve near-perfect success rates for individual patients is not adequately captured by the standard evaluation protocol, which measures attack success in aggregate across records. This remains true even when evaluating aggregate attack success at very low false-positive rates (for example, 10^−4^), which is the current standard practice. Thus, reporting standards for AI privacy audits need to change. Audits should report the success of privacy attacks at the level of individual data contributors or, if the necessary patient- or person-level identifiers are unavailable, at the record level.

We observed that the number of patients highly vulnerable to MIAs increases drastically for larger models. Although the magnitude of this change in patient-level risk was previously unknown, other works have also reported greater attack success against larger, more performant models^3,5,7,43^. This observation that privacy risks grow with model size and predictive performance is explained by theoretical research^44,45^, which postulates that, for long-tailed data distributions, fitting atypical records from the tail is necessary to achieve optimal performance on unseen data at test time. Our results provide further empirical support for this theory and, together, suggest that a trade-off between patient privacy and model performance is inevitable, particularly for rare diseases. Generally, as we found that the number of patients highly vulnerable to MIAs increases by orders of magnitude with larger models, we recommend carefully evaluating the need for the performance improvements they offer.

We found substantial differences in the frequency with which patients from different subgroups experience extreme MIA risk. The fact that some of these groups (for example, self-reported race subgroups in chest radiographs) are not readily distinguishable by human experts raises concerns that MIA risk differences, which probably exist beyond the stratification variables we investigated, may pass unnoticed in practice. We found that the observed risk differences are driven, at least in part, by group-size differences in the training data. Groups of patients that are underrepresented in a model training dataset are often overrepresented among the records most susceptible to MIAs. By contrast, the opposite often holds for majority groups. This finding—that a disproportionately large share of the AI privacy risk burden rests on underrepresented groups—complements the existing literature on health inequalities, which has reported worse health outcomes and life expectancy for marginalized and minority groups^46^. Our findings suggest that current trends in medical AI development and deployment could exacerbate these health inequalities. Previous research has shown that the diagnostic performance of AI models, which typically increases with the amount of suitable training data, can be significantly lower for underrepresented (minority) groups^36,37,47^. Thus, there is a possibility of a vicious cycle in which minority groups place decreasing levels of trust in AI model performance and security, leading to a decreased willingness to contribute to model training datasets.

MIAs facilitate data extraction attacks against generative AI models^5–7^. Thus, our findings have potentially far-reaching implications for generative AI privacy risk assessments. Extraction attacks allow for high-fidelity reconstruction of full individual records from the training dataset of a model and have been demonstrated for large language models^5^, diffusion-based image generation models^6^ and recently, aligned, production-level large language models^7^. Although our study focused on discriminative (diagnostic) AI models, the type of attack we studied is generally applicable and can be used against generative models with little to no modification. We thus see the exploration of our proposed methodology for estimating record- and patient-level MIA success against generative models as an interesting direction for future research. Given the substantial computational resources this would require, exploring scalable approximation techniques is another valuable avenue to investigate.

Unlocking the full potential of medical AI will require training models on vast medical datasets; this depends on gaining and upholding the trust of data-contributing patients. To this end, mathematically verifiable approaches to risk mitigation, such as DP^48^, are emerging as the most promising solution. DP, by carefully perturbing parameter updates with white noise during model training or fine-tuning^49^, limits the contribution of the data of any individual to the parameter update and, by extension, to the final model. This provably protects the privacy of any data-contributing patient, no matter how unique or atypical their data may be. Our experimental data confirmed that stronger levels of DP protection effectively reduce MIA success for all data-contributing patients. However, we also observed that mitigating MIAs requires stronger levels of DP protection than previously thought^3,50^. Specifically, our results indicate that fully mitigating MIAs for all data-contributing patients requires implementing DP protection at the patient level rather than at the record level. Recent research has demonstrated that, in practice, AI models can be trained with strong privacy guarantees while incurring minimal degradation in predictive performance compared with a non-private model^51–54^. We are thus optimistic that medical AI models protected by DP will have a significant positive impact on health outcomes globally without endangering the privacy of any data-contributing patient.

In summary, we present evidence that MIAs can be highly effective at compromising the privacy of individual data-contributing patients. Given this vulnerability, medical AI models and their deployment contexts should be assessed for the sensitive information that attackers could obtain by successfully inferring training dataset membership. To prevent privacy harm, we recommend that vulnerable models be protected by verifiable risk mitigation strategies and/or strict access controls.

## Methods

### CheXpert processing and model training details

CheXpert^26^ is a large chest radiograph dataset from Stanford Hospital comprising 224,313 chest radiographs from 64,539 patients. CheXpert comes with structured labels that were extracted automatically from free-text radiology reports and indicate the presence of 14 common thoracic conditions. The dataset provides demographic data that were obtained from the electronic data storage system of the hospital and includes information on the sex and age of the patient. Race (self-reported) and ethnicity (self-reported) data of the patient were released separately and can be matched by cross-linking patient IDs. Models for this dataset were trained with data augmentation (random horizontal flipping, random pixel shifts and random rotations) to detect the presence of all 14 conditions. We combined official CheXpert test and validation sets to accurately evaluate diagnostic model performance on a large set of expert-consensus-verified labels (total of *N* = 668 records). On this unseen data, a WRN-28-2 with around 1.5 million parameters trained on the records from a random 50% patient subset achieves a macro-average AUC score of 0.892 across the CheXpert competition classes: cardiomegaly, oedema, consolidation, atelectasis and pleural effusion.

### MIMIC-CXR processing and model training details

MIMIC-CXR^27^ is a large chest radiograph dataset consisting of 377,110 chest radiographs from 65,379 patients at the Beth Israel Deaconess Medical Center. We use MIMIC-CXR-JPG^55^, a subsequent re-release of the original dataset that contains images in ‘.jpg’ format and structured labels derived from free-text radiology reports. The structured labels indicate the presence of 14 common thoracic conditions and are in an identical format to the CheXpert dataset. The dataset comprises demographic data that were obtained from the electronic data storage system of the hospital and includes patients’ age, sex, race (self-reported), ethnicity (self-reported), marital status, insurance and mortality information. Some of the demographic data were released separately and can be matched by cross-linking patient identifiers. Models for this dataset were trained with data augmentation (random horizontal flipping, random pixel shifts and random rotations) to detect the presence of all 14 conditions. As expert-verified labels are not available for MIMIC-CXR, we evaluate model performance, as we did for CheXpert, on the combined set of records from the official CheXpert validation and test set (*N* = 668 records). On this unseen data, a WRN-28-2 with around 1.5 million parameters trained on the records from a random 50% patient subset achieves a macro-average AUC score of 0.851 across all classes.

### Fitzpatrick 17k processing and model training details

Fitzpatrick 17k^32^ is a dermatology dataset consisting of 16,523 RGB (red, green and blue) images from an unknown number of patients. Images in the dataset were initially sourced from two open-source dermatology atlases and complemented with Fitzpatrick skin-type labels, which were provided by trained non-experts. Owing to the noise expected in these labels, we grouped together types I/II, III/IV and V/VI, respectively. The dataset contains no demographic information apart from the Fitzpatrick skin-type labels. Models for this dataset were trained with data augmentation (random horizontal flipping and random pixel shifts) to classify images into one of three mutually exclusive classes (non-neoplastic, benign and malignant). Diagnostic performance was evaluated on the expert-verified test set (*N* = 348 records). On this unseen data, a WRN-28-2 with around 1.5 million parameters trained on the records from a random 50% patient subset achieves an accuracy of 0.746 and a macro-average AUC score of 0.872 across the three classes.

Note that patient identifiers are not available for Fitzpatrick 17k. Thus, to compute patient-level MIA AUC, we assume that each record belongs to a unique patient.

### FairVision processing and model training details

FairVision^56^ is an ophthalmoscopy dataset from the Department of Ophthalmoscopy at Harvard Medical School consisting of 30,000 scanning laser ophthalmoscopy fundus images from 30,000 unique patients. The dataset comprises demographic data that were obtained from the electronic data storage system of the hospital and includes patients’ age, gender, race (self-reported), ethnicity (self-reported), preferred language and marital status. The dataset consists of three patient cohorts, each with 10,000 patients or images and their respective disease labels indicating the presence or severity of age-related macular degeneration (AMD), diabetic retinopathy (DR) and glaucoma. To combine all available data into a single dataset for a seven-class classification problem, we merged the disease labels across the three cohorts under the assumption of mutual exclusivity (no patient from one cohort had a disease from another). The glaucoma label ‘no’, and AMD label ‘normal’ were merged to create the ‘healthy’ label. A basic image quality screening was performed, in which dark, low-contrast images with image-wise mean and s.d. below 1.0 and 3.0, respectively, were discarded. This left 28,542 images from 28,542 patients for model training. Models for this dataset were trained with data augmentation (random horizontal flipping, random pixel shifts) to classify images into one of seven mutually exclusive classes: healthy, non-vision-threatening DR, vision-threatening DR, glaucoma, early AMD, intermediate AMD and late AMD. Diagnostic performance was evaluated on *N* = 500 randomly chosen records from the official test set; the remaining images were used for model training. On these unseen data, a WRN-28-2 with around 1.5 million parameters trained on the records from a random 50% patient subset achieves a macro-average AUC score of 0.817 across all classes.

### EMBED processing and model training details

EMBED^38^ is a large mammography dataset consisting of 480,323 mammograms from 23,253 patients who underwent breast cancer screening or diagnosis at four hospital sites of Emory University in Atlanta. The dataset comprises demographic data that were obtained from electronic health records and includes patients’ age, race (self-reported), ethnicity (self-reported) and insurance status. We considered only craniocaudal and mediolateral oblique (MLO) views from screening exams and excluded any non-female patients, thus reducing the dataset size to 360,159 mammograms from 19,429 female patients. Following ref. ^57^, we train models to predict breast density, an important clinical risk factor for breast cancer. Models for this dataset were trained with data augmentation (random horizontal flipping and random pixel shifts) to classify images into one of the four BI-RADS breast density categories. Diagnostic performance was evaluated on a subset of 500 randomly picked patients (*N* = 11,121 images). On this unseen data, a WRN-28-2 with around 1.5 million parameters trained on the records from a random 50% patient subset achieves a macro-average AUC score of 0.936 across all classes.

### PTB-XL processing and model training details

PTB-XL^30^ is a cardiology dataset comprising 21,799 electrocardiograms from 18,869 patients. The dataset contains 12-lead electrocardiograms sampled at 100 Hz and 10 s long paired with their corresponding diagnostic label (multi-hot encoded), indicating the presence of common cardiac conditions. The dataset comprises demographic data obtained from the electronic data storage system of the hospital, including patients’ age and sex. Models for this dataset were trained without data augmentation to perform multi-label classification for the superclasses: normal, myocardial infarction, conduction disturbance, ST/T-changes and hypertrophy. Diagnostic performance was evaluated on the official test set. On these unseen data, a convolutional residual network^58^ with around 1.5 million parameters trained on the records from a random 50% patient subset achieves a macro-average AUC score of 0.916 across all classes.

### MIMIC-IV-ED processing and model training details

MIMIC-IV-ED^29^ is a large electronic health record dataset comprising data from 418,007 records from 201,213 patients at the emergency department of the Beth Israel Deaconess Medical Center. The dataset contains demographic data that were obtained from the electronic data storage system of the hospital and includes patients’ age, sex, race (self-reported), ethnicity (self-reported), marital status, insurance and mortality information. We followed the pre-processing steps in ref. ^59^ and trained models without data augmentation to predict hospitalization outcome (binary classification) based on 64 clinical features (discrete and continuous). Diagnostic performance was evaluated on the official test set in ref. ^59^. On these unseen data, a tabular residual network^60^ with around 1.5 million parameters trained on the records from a random 50% patient subset achieves an AUC score of 0.815 across all classes.

### General model training details

All investigated models were trained using stochastic gradient descent with momentum, exponential moving parameter average (EMA), weight decay, data augmentation and a learning rate schedule in the form of cosine decay with linear warm-up. Hyperparameter values were determined by a random search to maximize diagnostic performance (macro-average AUC) on unseen data. Specifically, for each dataset, a random search with *N* = 50 trials over suitable values for learning rate, weight decay, momentum and EMA decay was conducted (Supplementary Table 1). Training and test data were rescaled to have values within [−1, 1]. To prevent overfitting, model checkpointing was used, ensuring that only the model with the best generalization performance was retained after the training process terminated. We estimate that reproducing all of our experimental data would require around 900 h on a single A100 GPU.

### Strategies to improve training efficiency

Improving training efficiency was crucial because of the limited computational resources available to us. Thus, we resized all images to a spatial resolution of 64 × 64 pixels unless otherwise stated in the main text. Moreover, to further improve training efficiency, we used compilation caching with Keras^61^ JAX^62^ backend and used mixed-precision training with the default Keras policy: ‘mixed_float16’. Supplementary Table 1 shows training times of residual networks with about 1.5 million parameters for each of the investigated datasets using a single A100 GPU. Notably, the training runs required for our analysis are independent of one another, so the process can be easily accelerated by parallelizing them across multiple GPUs, with no need for inter-device communication during training.

### Adapting LR-MIAs to multi-label classification

LR-MIAs were designed for multi-class classification and are thus not directly compatible with multi-label classification settings in which multiple classes (diseases) may be present in a single input. We adapt LR-MIAs to the multi-label classification setting by using a softmax activation function on the raw network output (instead of the sigmoid function typically used). Then, to obtain a scalar quantity, we take the sum over the confidence values for all present classes (conditions). Note that our strategy does not change the assumed threat model. An attacker with access to the post-sigmoid activation confidence values can obtain the values necessary for our adapted attack by applying the inverse sigmoid (logit) function. Empirically, we found that our strategy performed better than estimating multivariate Gaussians for the confidence values of all present classes (the more natural adaptation of LR-MIAs to multi-label settings). This is probably because accurately estimating multivariate Gaussians requires more samples than their univariate counterparts.

### Estimating record-level LR-MIA success

Quantifying record-level MIA vulnerability requires measuring MIA performance independently for each target record across many target models. To do this with a manageable amount of computational resources, we leverage the fact that LR-MIAs are essentially hypothesis tests and directly estimate the empirical sampling distributions of the confidence of the target model under the null and alternative hypotheses. As these distributions are assumed to take Gaussian form in both LiRA^3^ and RMIA^4^, the record-level MIA AUC for a target record *x* admits a closed-form solution:

1 $$\hat{\theta }(x)=\varPhi \left(\frac{{\mu }_{x}-{\mu }_{{x}^{{\prime} }}}{\sqrt{{\sigma }_{x}^{2}+{\sigma }_{{x}^{{\prime} }}^{2}}}\right).$$

Here, *Φ* denotes the CDF of the standard normal and *μ*_*x*_, *μ*_*x*′_, ${\sigma }_{x}^{2}$, ${\sigma }_{{x}^{{\prime} }}^{2}$ denote sample means and variances of the logit-transformed confidence values provided by target models partitioned into models trained on *x* and models not trained on *x* (denoted by *x* and *x*′, respectively). To obtain a scalar quantity from the vector of predicted confidences for all possible classes, we follow the standard protocol^3,4^ and index the vector at the correct class indicated by the corresponding label. Multi-label confidence values were transformed, as explained in the previous section. Note that, for the Gaussian assumption of LR-MIAs to hold, confidence values need to be logit-transformed (${\rm{logit}}(p)\,=\,{\rm{ln}}\,\frac{p}{1-p}$). For models trained with data augmentation, we use *N* = 16 augmented queries to the target model; confidence values are then averaged across augmentations.

We calculate the standard error of $\widehat{\theta }$ (the bi-normal AUC) as described in ref. ^23^:

2 $$\mathrm{SE}(\hat{\theta }(x))=\sqrt{\frac{\hat{\theta }(x)(1-\hat{\theta }(x))+({N}_{{x}^{{\prime} }}-1)({Q}_{1}-\hat{\theta }(x))+({N}_{x}-1)({Q}_{2}-\hat{\theta }{(x)}^{2})}{{N}_{{x}^{{\prime} }}\times {N}_{x}}},$$

where $\widehat{\theta }(x)$ is the estimated AUC score from equation (1), ${Q}_{1}=\frac{\widehat{\theta }(x)}{1-\widehat{\theta }(x)}$ and ${Q}_{2}=\frac{2\widehat{\theta }{(x)}^{2}}{1+\widehat{\theta }(x)}$. *N*_*x*_ and ${N}_{{x}^{{\prime} }}$ refer to the number of target models trained and not trained on the target record, respectively. To ensure that each target model effectively reduces the standard error of the estimated AUC across all records, it is important to train the target models on random data subsets sampled in an unbiased fashion (see ref. ^3^ for details). This guarantees that *N*_*x*_ and ${N}_{{x}^{{\prime} }}$ are equal for all *x*.

Note that it is important to perform the subset sampling at the patient- rather than the record level to accurately estimate patient-level MIA vulnerability. This is to ensure that either all or none of a given patients’ records are included in a target model’s training dataset. In initial experiments, we sampled subsets at the record level and found that this led to underestimating MIA success for patients who contributed multiple similar records.

### Model scaling and differential privacy experiments

All models in the model scaling experiments were trained with the same hyperparameter values found during the initial hyperparameter tuning procedure described above. This decision was made because of the limited computational resources available to us.

Models for the DP experiments were trained with DP-Adam^49,63^ using the jax-privacy library^64^. Concretely, hyperparameters for these models were determined for a fixed privacy budget of *ε* = 100 using a random search with *N* = 20 trials over suitable values for the learning rate (see Supplementary Table 2 for results); EMA and weight decay were not used. The clipping threshold for DP-Adam was determined using the procedure described in ref. ^65^. Note that the diagnostic performance of the models could likely be improved by performing a separate hyperparameter search for each privacy budget and adapting the other engineering techniques proposed in ref. ^53^. Bounds on the MIA AUC implied by the record-level (*ε*, *δ*)-DP guarantee, where calculated from the trade-off curve obtained by converting the privacy profile of the sub-sampled Gaussian mechanism^66^ to f-DP^67^.

### Attacking open-source chest radiograph models

We follow the standard protocol as described in the original publication^4^ to conduct RMIA on the CheXpert and MIMIC-CXR models from TorchXrayVision. We use offline RMIA with a single pre-trained reference model (PadChest^25^, also from TorchXrayVision). Offline approximation parameter *a* was calculated as described in ref. ^4^; *a* = 0.857 and 0.918 for CheXpert and MIMIC-CXR, respectively. We did not use augmented queries for the target model; multi-label confidence values were transformed as described previously.

Notably, the open-source implementation of RMIA relies on a large outer product, which requires prohibitive amounts of memory when conducting the attack in parallel for many target records. Our adapted implementation greatly improves on this by holding only relevant entries of the large outer product matrix in memory, thereby easily scaling to large numbers of target records.

### Measuring MIA success with partial target records

We simulate settings in which MIAs are executed using only partial access to target records. Specifically, for MIMIC-IV-ED and PTB-XL, we train target models on random subsets of the full datasets and subsequently obtain confidence scores for these models when queried on partial target records, in which we redacted increasing amounts of information. We then compute record- and patient-level MIA AUC scores from confidence values as described previously. To redact information from target records, we simply set all relevant feature values to 0. For MIMIC-IV-ED, we investigate a setting we term ‘realistic subset’, in which all except the following 20 features are redacted: age, gender_binarized, triage_temperature, triage_heartrate, triage_resprate, triage_o2sat, triage_sbp, triage_dbp, triage_pain, triage_acuity, chiefcom_chest_pain, chiefcom_abdominal_pain, chiefcom_headache, chiefcom_shortness_of_breath, chiefcom_back_pain, chiefcom_cough, chiefcom_nausea_vomiting, chiefcom_fever_chills, chiefcom_syncope and chiefcom_dizziness. These features represent patients’ age, sex, chief complaints and vital signs at triage that we deem realistically obtainable for an adversary. We refer to Extended Data Table 1 from ref. ^59^ for a full description of the features in MIMIC-IV-ED. For MIMIC-IV-ED, we investigate another setting ‘most predictive’, in which all features except the following 10 features that ref. ^59^ identified as most predictive for hospitalization are redacted: age, triage_acuity, triage_sbp, triage_heartrate, triage_dbp, triage_temperature, triage_pain, triage_o2sat, triage_resprate and n_hosp_365d. For PTB-XL, we investigate settings in which an attacker has access to only temporal snippets of the 12-lead ECG signal and another setting in which only the signal from lead I is available. As for MIMIC-IV-ED, relevant information was redacted by replacing all signal values with zeros.

### Subgroup analysis

Our subgroup analysis compares the composition of the records most vulnerable to MIAs to that of the overall dataset. We measure *O*, the number of records observed for a given subgroup in the 99th record-level MIA AUC percentile and compare it with *E*, the expected number of records for this subgroup in the 99th percentile based on the composition of the overall training dataset. To this end, we compute the *χ*^2^ test statistic and Pearson residuals, which measure the contribution of a subgroup to the test statistic:

3 $$\mathrm{Pearson}\,\mathrm{residual}=\frac{O\,-\,E}{\sqrt{E}}.$$

The relative change between the observed and expected counts in the 99th percentile, as reported in the main body text, is calculated as

4 $${\varDelta }_{\mathrm{relative}}( \% )=\frac{(O\,-\,E)}{E}\times 100.$$

Note that we excluded records with incomplete (other/unknown) subgroup information from the subgroup analysis. Small discrepancies between Pearson residuals and data in Supplementary Tables 3–16 are thus to be expected.

### Reporting summary

Further information on research design is available in the Nature Portfolio Reporting Summary linked to this article.

## Online content

Any methods, additional references, Nature Portfolio reporting summaries, source data, extended data, supplementary information, acknowledgements, peer review information; details of author contributions and competing interests; and statements of data and code availability are available at 10.1038/s41586-026-10688-0.

## Supplementary information

Supplementary Information This file contains Supplementary Tables 1–16. Supplementary Tables 1 and 2 report hyperparameters for general model training and model training with differential privacy. The remaining tables report characteristics of the investigated datasets and the extreme MIA risk tails.

Reporting Summary

Peer Review File

## Data Availability

All datasets used in this study are publicly available for research purposes. See GitHub (https://github.com/moritzknolle/leakoscope) for access instructions.

## Code availability

Code and instructions to reproduce our results are available at GitHub (https://github.com/moritzknolle/leakoscope) and are permanently archived on Zenodo^68^ (10.5281/zenodo.20124226).
