# Supplementary material for: Disparate privacy risks from medical AI
Source: Nature. 2026 Jun 24;656(8126):192–8. doi: 10.1038/s41586-026-10688-0 (PMC13442007; doi:10.1038/s41586-026-10688-0)
Supplement: Supplementary file 1 [file 41586_2026_10688_MOESM1_ESM.pdf]

nature portfolio

https://doi.org/10.1038/s41586-026-10688-0

Supplementary information

Disparate privacy risks from medical AI

In the format provided by the authors and unedited

Supplementary Material

List of Tables

1 Model training hyperparameters. 2
2 DP training hyperparameters. 3
3 Characteristics of the CheXpert dataset. 4
4 Characteristics of the MIMIC-CXR dataset. 5
5 Characteristics of the Fitzpatrick 17k dataset. 6
6 Characteristics of the Fairvision dataset. 7
7 Characteristics of the EMBED dataset. 8
8 Characteristics of the PTB-XL dataset. 9
9 Characteristics of the MIMIC-IV-ED dataset. 10
10 Characteristics of the extreme MIA risk tail in the CheXpert dataset. 11
11 Characteristics of the extreme MIA risk tail in the MIMIC-CXR dataset. 12
12 Characteristics of the extreme MIA risk tail in Fitzpatrick 17k dataset. 13
13 Characteristics of the extreme MIA risk tail in the Fairvision dataset. 14
14 Characteristics of the extreme MIA risk tail in the EMBED dataset. 15
15 Characteristics of the extreme MIA risk tail in the PTB-XL dataset. 16
16 Characteristics of the extreme MIA risk tail in the MIMIC-IV-ED dataset. 17

**Dataset**	α	λ	μ	γ	**Batch size**	**Epochs**	**Time (min)**
CheXpert	1.0	10^{-4}	0.9	0.9995	1024	60	12
MIMIC-CXR	0.25	10^{-3}	0.99	0.95	1024	60	20
Fitzpatrick-17k	5×10^{-3}	10^{-4}	0.9	0.99	256	100	3
FairVision	10^{-4}	10^{-6}	0.9	0.99	256	100	3
EMBED	10^{-1}	10^{-8}	0.9	0.995	1024	30	8
PTB-XL	0.25	5×10^{-3}	0.9	0.995	1024	100	4
MIMIC-IV-ED	10^{-1}	10^{-7}	0.9	0.995	1024	100	3

Table 1: Model training hyperparameters. α, λ, μ and γ denote learning rate, weight decay, momentum, and EMA decay rate, respectively. Training times were measured on a single A100 GPU.

**Dataset**	α	λ	**C**	**Batch size**	**Epochs**	**Time (min)**
EMBED	3×10^{-3}	0.0	$10,000$	512	20	30
PTB-XL	5×10^{-3}	0.0	$50,000$	1024	200	7

Table 2: DP training hyperparameters. α, λ, and C denote learning rate, weight decay and clipping threshold, respectively. Training times were measured on a single A100 GPU.

		All	Asian	Black	White	Other
	**Patients**	64,539	6,936	3,093	36,110	18,400
	**Records**	223,413	23,272	11,961	125,491	62,689
	**Median Age (s.d.)**	62 (18)	63 (17)	56 (17)	64 (17)	57 (18)
**Sex**	Female (%)	90,777 (40.6)	9,958 (42.8)	5,839 (48.8)	49,504 (39.4)	25,476 (40.6)
	Male (%)	132,636 (59.4)	13,314 (57.2)	6,122 (51.2)	75,987 (60.6)	37,213 (59.4)
**Imaging Protocol**	AP (%)	161,590 (72.3)	16,292 (70.0)	8,387 (70.1)	91,254 (72.7)	45,657 (72.8)
	PA (%)	29,419 (13.2)	3,344 (14.4)	1,664 (13.9)	1,6234 (12.9)	8,177 (13.0)
	Other (%)	32,404 (14.5)	3,636 (15.6)	1,910 (16.0)	18,003 (14.3)	8,855 (14.1)
**Diagnostic Label**	No Finding (%)	22,380 (10.0)	2,412 (10.4)	1,398 (11.7)	11,786 (9.4)	6,784 (10.8)
	Enlarged Cardiomediastinum (%)	10,798 (4.8)	1,123 (4.8)	593 (5.0)	6,131 (4.9)	2,951 (4.7)
	Cardiomegaly (%)	27,000 (12.1)	2,943 (12.6)	2,338 (19.5)	14,394 (11.5)	7,325 (11.7)
	Lung Opacity (%)	105,581 (47.3)	11,264 (48.4)	5,380 (45.0)	60,369 (48.1)	28,568 (45.6)
	Lung Lesion (%)	9,186 (4.1)	1,422 (6.1)	379 (3.2)	4,941 (3.9)	2,444 (3.9)
	Edema (%)	52,246 (23.4)	4,600 (19.8)	3,046 (25.5)	29,198 (23.3)	15,402 (24.6)
	Consolidation (%)	14,783 (6.6)	1,708 (7.3)	690 (5.8)	8,243 (6.6)	4,142 (6.6)
	Pneumonia (%)	6,039 (2.7)	686 (2.9)	359 (3.0)	3,354 (2.7)	1,640 (2.6)
	Atelectasis (%)	33,376 (14.9)	3,077 (13.2)	1,479 (12.4)	18,886 (15.0)	9,934 (15.8)
	Pneumothorax (%)	19,448 (8.7)	2,279 (9.8)	687 (5.7)	11,441 (9.1)	5,041 (8.0)
	Pleural Effusion (%)	86,187 (38.6)	9,414 (40.5)	3,791 (31.7)	49,438 (39.4)	23,544 (37.6)
	Pleural Other (%)	3,523 (1.6)	393 (1.7)	133 (1.1)	2,120 (1.7)	877 (1.4)
	Fracture (%)	9,040 (4.0)	747 (3.2)	292 (2.4)	5,588 (4.5)	2,413 (3.8)
	Support Devices (%)	116,001 (51.9)	11,611 (49.9)	5,583 (46.7)	65,297 (52.0)	33,510 (53.5)

Table 3: Characteristics of the CheXpert dataset.

		All	Asian	Black	White	Other
	**Patients**	65,379	1,637	8,051	30,941	24,750
	**Records**	377,110	8,911	50,750	197,205	120,244
**Sex**	Female (%)	170,803 (45.3)	3,862 (43.3)	30,792 (60.7)	87,711 (44.5)	48,438 (40.3)
	Male (%)	186,739 (49.5)	5,049 (56.7)	19,958 (39.3)	109,494 (55.5)	52,238 (43.4)
**Imaging Protocol**	PA (%)	96,161 (25.5)	2,301 (25.8)	14,300 (28.2)	45,739 (23.2)	33,821 (28.1)
	L (%)	82,853 (22.0)	1,752 (19.7)	14,189 (28.0)	37,598 (19.1)	29,314 (24.4)
	AP (%)	147,173 (39.0)	3,523 (39.5)	17,303 (34.1)	84,344 (42.8)	42,003 (34.9)
	LL (%)	35,133 (9.3)	1,027 (11.5)	4,097 (8.1)	20,088 (10.2)	9,921 (8.3)
	Other (%)	15,790 (4.2)	308 (3.5)	861 (1.7)	9,436 (4.8)	5,185 (4.3)
**Diagnostic Label**	No Finding (%)	149,986 (39.8)	3,286 (36.9)	22,643 (44.6)	68,604 (34.8)	55,453 (46.1)
	Enlarged Cardiomediastinum (%)	9,847 (2.6)	292 (3.3)	1,072 (2.1)	5,704 (2.9)	2,779 (2.3)
	Cardiomegaly (%)	56,480 (15.0)	1,410 (15.8)	8,845 (17.4)	29,989 (15.2)	16,236 (13.5)
	Lung Opacity (%)	76,213 (20.2)	1,877 (21.1)	9,299 (18.3)	43,338 (22.0)	21,699 (18.0)
	Lung Lesion (%)	10,591 (2.8)	369 (4.1)	1,233 (2.4)	6,119 (3.1)	2,870 (2.4)
	Edema (%)	36,070 (9.6)	691 (7.8)	4,608 (9.1)	20,520 (10.4)	10,251 (8.5)
	Consolidation (%)	14,420 (3.8)	404 (4.5)	1,624 (3.2)	8,386 (4.3)	4,006 (3.3)
	Pneumonia (%)	25,115 (6.7)	596 (6.7)	3096 (6.1)	13,954 (7.1)	7,469 (6.2)
	Atelectasis (%)	65,477 (17.4)	1,568 (17.6)	7,160 (14.1)	38,642 (19.6)	18,107 (15.1)
	Pneumothorax (%)	12,853 (3.4)	463 (5.2)	917 (1.8)	7,985 (4.0)	3,488 (2.9)
	Pleural Effusion (%)	75,613 (20.1)	2,091 (23.5)	6,843 (13.5)	47,167 (23.9)	19,512 (16.2)
	Pleural Other (%)	3,450 (0.9)	104 (1.2)	321 (0.6)	2,116 (1.1)	909 (0.8)
	Fracture (%)	6,341 (1.7)	93 (1.0)	384 (0.8)	3,976 (2.0)	1,888 (1.6)
	Support Devices (%)	83,075 (22.0)	2,114 (23.7)	8,345 (16.4)	48,392 (24.5)	24,224 (20.1)

Table 4: Characteristics of the MIMIC-CXR dataset.

		All	Type I/II	Type III/IV	Type V/VI
	**Records**	16,523	7,737	6,072	2,155
**Diagnostic Label (coarse)**	Non-neoplastic (%)	12,032 (72.8)	5,429 (70.2)	4,473 (73.7)	1,746 (81.0)
	Benign (%)	2,231 (13.5)	1,113 (14.4)	842 (13.9)	203 (9.4)
	Malignant (%)	2,260 (13.7)	1,195 (15.4)	757 (12.5)	206 (9.6)
**Diagnostic Label (fine)**	Inflammatory (%)	10,838 (65.6)	5,025 (64.9)	3,956 (65.2)	1,502 (69.7)
	Benign Dermal (%)	1,067 (6.5)	580 (7.5)	361 (5.9)	77 (3.6)
	Malignant Dermal (%)	156 (0.9)	44 (0.6)	71 (1.2)	36 (1.7)
	Malignant Melanoma (%)	570 (3.4)	331 (4.3)	168 (2.8)	45 (2.1)
	Genodermatoses (%)	1,194 (7.2)	404 (5.2)	517 (8.5)	244 (11.3)
	Malignant Epidermal (%)	1,352 (8.2)	732 (9.5)	447 (7.4)	108 (5.0)
	Benign Epidermal (%)	931 (5.6)	407 (5.3)	400 (6.6)	102 (4.7)
	Benign Melanocyte (%)	233 (1.4)	126 (1.6)	81 (1.3)	24 (1.1)
	Malignant Cutaneous Lymphoma (%)	182 (1.1)	88 (1.1)	71 (1.2)	17 (0.8)

Table 5: Characteristics of the Fitzpatrick 17k dataset.

		All	Asian	Black	White
	**Patients**	28,542	2,141	3,683	22,718
	**Records**	28,542	2,141	3,683	22,718
	**Median Age (s.d.)**	67 (17)	62 (18)	62 (16)	68 (17)
**Sex**	Female (%)	16,419 (57.5)	1,159 (54.1)	2,094 (56.9)	13,166 (58.0)
	Male (%)	12,123 (42.5)	982 (45.9)	1,589 (43.1)	9,552 (42.0)
**Diagnostic Label**	Healthy (%)	11,041 (38.7)	932 (43.5)	1,474 (40.0)	8,635 (38.0)
	Glaucoma (%)	4,569 (16.0)	356 (16.6)	825 (22.4)	3,388 (14.9)
	Non-vision threatening DR (%)	8,687 (30.4)	657 (30.7)	1,135 (30.8)	6,895 (30.4)
	Vision threatening DR (%)	816 (2.9)	48 (2.2)	173 (4.7)	595 (2.6)
	Early AMD (%)	863 (3.0)	62 (2.9)	32 (0.9)	769 (3.4)
	Intermediate AMD (%)	1,144 (4.0)	33 (1.5)	19 (0.5)	1,092 (4.8)
	Late AMD (%)	1,422 (5.0)	53 (2.5)	25 (0.7)	1,344 (5.9)

Table 6: Characteristics of the Fairvision dataset.

		**All**	**Asian**	**Black**	**White**	**Other**
**Patients**		19,429	1,299	8,120	7,896	2,114
**Records**		360,159	20,819	162,012	152,965	24,363
**Median Age (s.d.)**		60 (12)	53 (11)	59 (11)	62 (12)	53 (10)
**Sex**	Female (%)	360,159 (100.0)	20,819 (100.0)	162,012 (100.0)	152,965 (100.0)	24,363 (100.0)
**BI-RADS Density**	A (%)	41,899 (11.6)	474 (2.3)	25,813 (15.9)	13,900 (9.1)	1,712 (7.0)
	B (%)	153,523 (42.6)	5,207 (25.0)	75,606 (46.7)	63,246 (41.3)	9,464 (38.8)
	C (%)	146,108 (40.6)	12,247 (58.8)	54,524 (33.7)	67,658 (44.2)	11,679 (47.9)
	D (%)	18,629 (5.2)	2,891 (13.9)	6,069 (3.7)	8,161 (5.3)	1,508 (6.2)
**BI-RADS Score**	0 (%)	54,743 (15.2)	3,809 (18.3)	2,4423 (15.1)	20,898 (13.7)	5,613 (23.0)
	1 (%)	271,205 (75.3)	15,669 (75.3)	123,004 (75.9)	115,426 (75.5)	17,106 (70.2)
	2 (%)	34,046 (9.5)	1,335 (6.4)	14,540 (9.0)	16,564 (10.8)	1,607 (6.6)
	3 (%)	19 (0.0)	6 (0.0)	7 (0.0)	6 (0.0)	0 (0.0)
	4 (%)	95 (0.0)	0 (0.0)	33 (0.0)	54 (0.0)	8 (0.0)
	5 (%)	9 (0.0)	0 (0.0)	0 (0.0)	9 (0.0)	0 (0.0)
	6 (%)	42 (0.0)	0 (0.0)	5 (0.0)	8 (0.0)	29 (0.1)
**Imaging Protocol**	CC (%)	171,025 (47.5)	10,047 (48.3)	76,550 (47.2)	72,891 (47.7)	11,537 (47.4)
	MLO (%)	189,134 (52.5)	10,772 (51.7)	85,462 (52.8)	80,074 (52.3)	12,826 (52.6)
**Image Type**	2D (%)	256,730 (71.3)	14,075 (67.6)	112,710 (69.6)	11,1813 (73.1)	18,132 (74.4)
	C-view (%)	103,429 (28.7)	6,744 (32.4)	49,302 (30.4)	41,152 (26.9)	6,231 (25.6)

Table 7: Characteristics of the EMBED dataset.

		All	Female	Male
	**Patients**	18,869	9,640	9,229
	**Records**	21,799	11,354	10,445
	**Median Age (s.d.)**	62 (32)	61 (24)	64 (39)
**Extra Systole**	Yes (%)	1,949 (8.9)	1,127 (9.9)	822 (7.9)
	No (%)	19,850 (91.1)	10,227 (90.1)	9,623 (92.1)
**Diagnostic Label**	Normal (%)	9,514 (43.6)	4,386 (38.6)	5,128 (49.1)
	Myocardial Infarction (%)	5,469 (25.1)	3,407 (30.0)	2,062 (19.7)
	ST/T-changes (%)	5,235 (24.0)	2,566 (22.6)	2,669 (25.6)
	Conduction Disturbance (%)	4,898 (22.5)	2,999 (26.4)	1,899 (18.2)
	Hypertrophy (%)	2,649 (12.2)	1,520 (13.4)	1,129 (10.8)

Table 8: Characteristics of the PTB-XL dataset.

		**All**	**Asian**	**Black**	**White**	**Other**
**Patients**		201,213	11,373	32,742	126,273	33,970
**Records**		418,007	18,319	92,151	242,617	64,920
**Sex**	Female (%)	226,968 (54.3)	10,247 (55.9)	55,690 (60.4)	125,324 (51.7)	35,707 (55.0)
	Male (%)	191,039 (45.7)	8,072 (44.1)	36,461 (39.6)	117,293 (48.3)	29,213 (45.0)
**Obesity**	Yes (%)	36,850 (8.8)	181 (1.0)	11,262 (12.2)	20,540 (8.5)	4,867 (7.5)
	No (%)	381157 (91.2)	18,138 (99.0)	80,889 (87.8)	22,2077 (91.5)	60,053 (92.5)
**Cancer**	Yes (%)	26,993 (6.5)	1,180 (6.4)	4,964 (5.4)	18,097 (7.5)	2,752 (4.2)
	No (%)	391014 (93.5)	17,139 (93.6)	87,187 (94.6)	224,520 (92.5)	62,168 (95.8)
**Hospitalised**	Yes (%)	197,774 (47.3)	7,122 (38.9)	36,220 (39.3)	129,189 (53.2)	25,243 (38.9)
	No (%)	220,233 (52.7)	11,197 (61.1)	55,931 (60.7)	113,428 (46.8)	39,677 (61.1)
**Insurance**	Medicare (%)	77,251 (18.5)	1,375 (7.5)	12,030 (13.1)	57,204 (23.6)	6,642 (10.2)
	Medicaid (%)	17,491 (4.2)	871 (4.8)	5,032 (5.5)	7,592 (3.1)	3,996 (6.2)
	Other (%)	32,3265 (77.3)	16,073 (87.7)	75,089 (81.5)	177,821 (73.3)	54,282 (83.6)

Table 9: Characteristics of the MIMIC-IV-ED dataset.

		All	Asian	Black	White	Other/Unknown
	**Patients**	1,233	148	81	651	353
	**Records**	2,234	254	166	1,081	733
	**Median Age (s.d.)**	57 (19)	57 (19)	53 (15)	60 (19)	52 (19)
**Sex**	Female (%)	858 (38.4)	102 (40.2)	98 (59.0)	412 (38.1)	246 (33.6)
	Male (%)	1,376 (61.6)	152 (59.8)	68 (41.0)	669 (61.9)	487 (66.4)
**Imaging Protocol**	AP (%)	1,355 (60.7)	151 (59.4)	107 (64.5)	612 (56.6)	485 (66.2)
	PA (%)	327 (14.6)	41 (16.1)	17 (10.2)	181 (16.7)	88 (12.0)
	Other (%)	552 (24.7)	62 (24.4)	42 (25.3)	288 (26.6)	160 (21.8)
**Diagnostic Label**	No Finding (%)	235 (10.5)	23 (9.1)	13 (7.8)	128 (11.8)	71 (9.7)
	Enlarged Cardiomediastinum (%)	105 (4.7)	16 (6.3)	8 (4.8)	47 (4.3)	34 (4.6)
	Cardiomegaly (%)	369 (16.5)	52 (19.8)	59 (34.9)	151 (14.0)	107 (14.8)
	Lung Opacity (%)	1,097 (49.1)	141 (55.5)	77 (46.4)	521 (48.2)	358 (48.8)
	Lung Lesion (%)	104 (4.7)	23 (9.1)	4 (2.4)	46 (4.3)	31 (4.2)
	Edema (%)	358 (16.0)	39 (15.4)	53 (31.9)	153 (14.2)	113 (15.4)
	Consolidation (%)	134 (6.0)	10 (3.9)	10 (6.0)	58 (5.4)	56 (7.6)
	Pneumonia (%)	56 (2.5)	6 (2.4)	5 (3.0)	32 (3.0)	13 (1.8)
	Atelectasis (%)	230 (10.3)	25 (9.8)	13 (7.8)	122 (11.3)	70 (9.5)
	Pneumothorax (%)	575 (25.7)	59 (23.2)	36 (21.7)	281 (26.0)	199 (27.1)
	Pleural Effusion (%)	721 (32.3)	113 (44.5)	50 (30.1)	323 (29.9)	235 (32.1)
	Pleural Other (%)	59 (2.6)	9 (3.5)	2 (1.2)	36 (3.3)	12 (1.6)
	Fracture (%)	92 (4.1)	19 (7.5)	2 (1.2)	38 (3.5)	33 (4.5)
	Support Devices (%)	1,240 (55.5)	128 (50.4)	88 (53.0)	601 (55.6)	423 (57.7)

Table 10: Characteristics of the extreme MIA risk tail in the CheXpert dataset.

		All	Asian	Black	White	Other
	**Patients**	954	29	146	576	203
	**Records**	3,494	114	523	2,154	703
**Sex**	Female (%)	1,372 (39.3)	13 (11.4)	293 (56.0)	798 (37.0)	268 (38.1)
	Male (%)	2,122 (60.7)	101 (88.6)	230 (44.0)	1,356 (63.0)	435 (61.9)
**Imaging Protocol**	PA (%)	1,084 (31.0)	20 (17.5)	178 (34.0)	711 (33.0)	175 (24.9)
	L (%)	759 (21.7)	16 (14.0)	162 (31.0)	397 (18.4)	184 (26.2)
	AP (%)	1,126 (32.2)	58 (50.9)	145 (27.7)	684 (31.8)	239 (34.0)
	LL (%)	420 (12.0)	19 (16.7)	34 (6.5)	301 (14.0)	66 (9.4)
	Other (%)	105 (3.0)	1 (0.9)	4 (0.8)	61 (2.8)	39 (5.5)
**Diagnostic Label**	No Finding (%)	638 (18.3)	15 (13.2)	162 (31.0)	387 (18.0)	74 (10.5)
	Enlarged Cardiomediastinum (%)	107 (3.1)	6 (5.3)	13 (2.5)	52 (2.4)	36 (5.1)
	Cardiomegaly (%)	405 (11.6)	8 (7.0)	100 (19.1)	185 (8.6)	112 (15.9)
	Lung Opacity (%)	848 (24.3)	31 (27.2)	141 (27.0)	491 (22.8)	185 (26.3)
	Lung Lesion (%)	273 (7.8)	10 (8.8)	36 (6.9)	146 (6.8)	81 (11.5)
	Edema (%)	243 (7.0)	1 (0.9)	60 (11.5)	125 (5.8)	57 (8.1)
	Consolidation (%)	199 (5.7)	7 (6.1)	25 (4.8)	116 (5.4)	51 (7.3)
	Pneumonia (%)	291 (8.3)	7 (6.1)	29 (5.5)	203 (9.4)	52 (7.4)
	Atelectasis (%)	466 (13.3)	8 (7.0)	50 (9.6)	326 (15.1)	82 (11.7)
	Pneumothorax (%)	1,292 (37.0)	62 (54.4)	87 (16.6)	907 (42.1)	236 (33.6)
	Pleural Effusion (%)	386 (11.0)	13 (11.4)	54 (10.3)	247 (11.5)	72 (10.2)
	Pleural Other (%)	61 (1.7)	2 (1.8)	2 (0.4)	43 (2.0)	14 (2.0)
	Fracture (%)	55 (1.6)	0 (0.0)	0 (0.0)	41 (1.9)	14 (2.0)
	Support Devices (%)	1,001 (28.6)	43 (37.7)	99 (18.9)	694 (32.2)	165 (23.5)

Table 11: Characteristics of the extreme MIA risk tail in the MIMIC-CXR dataset.

		All	Type I/II	Type III/IV	Type V/VI
**Records**		161	61	64	22
**Diagnostic Label (coarse)**	Non-neoplastic (%)	7 (4.3)	2 (3.3)	1 (1.6)	2 (9.1)
	Benign (%)	40 (24.8)	13 (21.3)	17 (26.6)	7 (31.8)
	Malignant (%)	114 (70.8)	46 (75.4)	46 (71.9)	13 (59.1)
**Diagnostic Label (fine)**	Inflammatory (%)	6 (3.7)	2 (3.3)	1 (1.6)	2 (9.1)
	Benign dermal (%)	28 (17.4)	11 (18.0)	9 (14.1)	5 (22.7)
	Malignant dermal (%)	8 (5.0)	0 (0.0)	3 (4.7)	3 (13.6)
	Malignant melanoma (%)	31 (19.3)	14 (23.0)	13 (20.3)	3 (13.6)
	Genodermatoses (%)	1 (0.6)	0 (0.0)	0 (0.0)	0 (0.0)
	Malignant epidermal (%)	66 (41.0)	28 (45.9)	26 (40.6)	6 (27.3)
	Benign epidermal (%)	7 (4.3)	1 (1.6)	5 (7.8)	1 (4.5)
	Benign melanocyte (%)	5 (3.1)	1 (1.6)	3 (4.7)	1 (4.5)
	Malignant cutaneous lymphoma (%)	9 (5.6)	4 (6.6)	4 (6.2)	1 (4.5)

Table 12: Characteristics of the extreme MIA risk tail in Fitzpatrick 17k dataset.

		All	Asian	Black	White
	**Patients**	199	12	17	170
	**Records**	199	12	17	170
	**Median Age (s.d.)**	73 (17)	72 (14)	65 (9)	73 (17)
**Sex**	Female (%)	128 (64.3)	7 (58.3)	7 (41.2)	114 (67.1)
	Male (%)	71 (35.7)	5 (41.7)	10 (58.8)	56 (32.9)
**Diagnostic Label**	Healthy (%)	20 (10.1)	1 (8.3)	4 (23.5)	15 (8.8)
	Glaucoma (%)	18 (9.0)	2 (16.7)	4 (23.5)	12 (7.1)
	Vision threatening DR (%)	37 (18.6)	4 (33.3)	6 (35.3)	27 (15.9)
	Non-vision threatening DR (%)	24 (12.1)	1 (8.3)	0 (0.0)	23 (13.5)
	Early AMD (%)	28 (14.1)	2 (16.7)	2 (11.8)	24 (14.1)
	Intermediate AMD (%)	32 (16.1)	1 (8.3)	1 (5.9)	30 (17.6)
	Late AMD (%)	40 (20.1)	1 (8.3)	0 (0.0)	39 (22.9)

Table 13: Characteristics of the extreme MIA risk tail in the Fairvision dataset.

		**All**	**Asian**	**Black**	**White**	**Other**
**Patients**		416	20	189	191	16
**Records**		3,512	142	1,780	1,497	93
**Median Age (s.d.)**		63 (11)	60 (12)	62 (11)	63 (12)	48 (9)
**Sex**	Female (%)	3,512 (100.0)	142 (100.0)	1,780 (100.0)	1,497 (100.0)	93 (100.0)
**BI-RADS Density**	A (%)	782 (22.3)	0 (0.0)	393 (22.1)	370 (24.7)	19 (20.4)
	B (%)	586 (16.7)	11 (7.7)	253 (14.2)	297 (19.8)	25 (26.9)
	C (%)	594 (16.9)	23 (16.2)	341 (19.2)	220 (14.7)	10 (10.8)
	D (%)	1,550 (44.1)	108 (76.1)	793 (44.6)	610 (40.7)	39 (41.9)
**BI-RADS Score**	0 (%)	321 (9.1)	13 (8.5)	164 (9.3)	125 (8.3)	19 (21.1)
	1 (%)	2,649 (75.4)	100 (70.4)	1,361 (76.5)	1,146 (76.6)	42 (45.2)
	2 (%)	537 (15.3)	29 (20.4)	251 (14.1)	227 (15.2)	30 (32.3)
	3 (%)	0 (0.0)	0 (0.0)	0 (0.0)	0 (0.0)	0 (0.0)
	4 (%)	11 (0.3)	0 (0.0)	11 (0.6)	0 (0.0)	0 (0.0)
	5 (%)	0 (0.0)	0 (0.0)	0 (0.0)	0 (0.0)	0 (0.0)
	6 (%)	0 (0.0)	0 (0.0)	0 (0.0)	0 (0.0)	0 (0.0)
**Imaging Protocol**	CC (%)	1,507 (42.9)	74 (52.1)	780 (43.8)	618 (41.3)	35 (37.6)
	MLO (%)	2,005 (57.1)	68 (47.9)	1,000 (56.2)	879 (58.7)	58 (62.4)
**Image Type**	2D (%)	2,489 (70.9)	97 (68.3)	1,201 (67.5)	1,116 (74.5)	75 (80.6)
	C-view (%)	1,023 (29.1)	45 (31.7)	579 (32.5)	381 (25.5)	18 (19.4)

Table 14: Characteristics of the extreme MIA risk tail in the EMBED dataset.

		All	Female	Male
	**Patients**	171	90	81
	**Records**	196	112	84
	**Median Age (s.d.)**	65 (41)	63 (28)	66 (53)
**Extra Systole**	Yes (%)	25 (12.8)	17 (15.2)	8 (9.5)
	No (%)	171 (87.2)	95 (84.8)	76 (90.5)
**Diagnostic Label**	Normal (%)	56 (28.6)	32 (28.6)	24 (28.6)
	Myocardial infarction (%)	62 (31.6)	36 (32.1)	26 (31.0)
	ST/T-changes (%)	51 (26.0)	24 (21.4)	27 (32.1)
	Conduction disturbance (%)	54 (27.6)	39 (34.8)	15 (17.9)
	Hypertrophy (%)	20 (10.2)	13 (11.6)	7 (8.3)

Table 15: Characteristics of the extreme MIA risk tail in the PTB-XL dataset.

		**All**	**Asian**	**Black**	**White**	**Other**
	**Patients**	2,171	91	502	1,278	310
	**Records**	3,344	98	985	1,862	399
**Sex**	Female (%)	1,731 (51.8)	51 (52.0)	469 (47.6)	983 (52.8)	228 (57.1)
	Male (%)	1,613 (48.2)	47 (48.0)	516 (52.4)	879 (47.2)	171 (42.9)
**Obesity**	Yes (%)	464 (13.9)	2 (2.0)	162 (16.4)	252 (13.5)	48 (12.0)
	No (%)	2,880 (86.1)	96 (98.0)	823 (83.6)	1,610 (86.5)	351 (88.0)
**Cancer**	Yes (%)	240 (7.2)	11 (11.2)	62 (6.3)	142 (7.6)	25 (6.3)
	No (%)	3,104 (92.8)	87 (88.8)	923 (93.7)	1,720 (92.4)	374 (93.7)
**Hospitalised**	Yes (%) 1,763 (52.7)	43 (43.9)	561 (57.0)	1,009 (54.2)	150 (37.6)	
	No (%)	1,581 (47.3)	55 (56.1)	424 (43.0)	853 (45.8)	249 (62.4)
**Insurance**	Medicare (%)	519 (15.5)	4 (4.1)	127 (12.9)	351 (18.9)	37 (9.3)
	Medicaid (%)	333 (10.0)	5 (5.1)	180 (18.3)	125 (6.7)	23 (5.8)
	Other (%)	2,492 (74.5)	89 (90.8)	678 (68.8)	1,386 (74.4)	339 (85.0)

Table 16: Characteristics of the extreme MIA risk tail in the MIMIC-IV-ED dataset.
