# Supplementary material for: Disparate privacy risks from medical AI
Source: Nature. 2026 Jun 24;656(8126):192–8. doi: 10.1038/s41586-026-10688-0 (PMC13442007; doi:10.1038/s41586-026-10688-0)
Supplement: Supplementary file 2 [file 41586_2026_10688_MOESM2_ESM.pdf]

nature portfolio

Corresponding author(s): Moritz Knolle

Last updated by author(s): May 11, 2026

Reporting Summary

Nature Portfolio wishes to improve the reproducibility of the work that we publish. This form provides structure for consistency and transparency in reporting. For further information on Nature Portfolio policies, see our Editorial Policies and the Editorial Policy Checklist.

Statistics

For all statistical analyses, confirm that the following items are present in the figure legend, table legend, main text, or Methods section.

n/a	Confirmed	
☐	☒	The exact sample size (*n*) for each experimental group/condition, given as a discrete number and unit of measurement
☐	☒	A statement on whether measurements were taken from distinct samples or whether the same sample was measured repeatedly
☐	☒	The statistical test(s) used AND whether they are one- or two-sided<br>*Only common tests should be described solely by name; describe more complex techniques in the Methods section.*
☐	☒	A description of all covariates tested
☐	☒	A description of any assumptions or corrections, such as tests of normality and adjustment for multiple comparisons
☐	☒	A full description of the statistical parameters including central tendency (e.g. means) or other basic estimates (e.g. regression coefficient) AND variation (e.g. standard deviation) or associated estimates of uncertainty (e.g. confidence intervals)
☐	☒	For null hypothesis testing, the test statistic (e.g. *F*, *t*, *r*) with confidence intervals, effect sizes, degrees of freedom and *P* value noted<br>*Give P values as exact values whenever suitable.*
☒	☐	For Bayesian analysis, information on the choice of priors and Markov chain Monte Carlo settings
☒	☐	For hierarchical and complex designs, identification of the appropriate level for tests and full reporting of outcomes
☐	☒	Estimates of effect sizes (e.g. Cohen's *d*, Pearson's *r*), indicating how they were calculated

Our web collection on statistics for biologists contains articles on many of the points above.

Software and code

Policy information about availability of computer code

Data collection	No software was used for data collection.
Data analysis	Code and instructions to reproduce our results are available on GitHub (https://github.com/moritzknolle/leakoscope) and are permanently archived on Zenodo (https://doi.org/10.5281/zenodo.20124226).

For manuscripts utilizing custom algorithms or software that are central to the research but not yet described in published literature, software must be made available to editors and reviewers. We strongly encourage code deposition in a community repository (e.g. GitHub). See the Nature Portfolio guidelines for submitting code & software for further information.

Data

Policy information about availability of data

All manuscripts must include a data availability statement. This statement should provide the following information, where applicable:

- Accession codes, unique identifiers, or web links for publicly available datasets
- A description of any restrictions on data availability
- For clinical datasets or third party data, please ensure that the statement adheres to our policy

All datasets used in this study are publicly available for research purposes. See GitHub (https://github.com/moritzknolle/leakoscope) for access instructions.

Research involving human participants, their data, or biological material

Policy information about studies with human participants or human data. See also policy information about sex, gender (identity/presentation), and sexual orientation and race, ethnicity and racism.

Reporting on sex and gender	We use sex information to investigate subgroup privacy risk differences. This information is included in the original datasets and was derived from electronic hospital records.
Reporting on race, ethnicity, or other socially relevant groupings	We use self-reported race information to investigate subgroup privacy risk differences. This information is included in the original datasets and was derived from electronic hospital records.
Population characteristics	Population characteristics differ for each of the investigated datasets, Supplementary Tables 3-10 provide summary information about each dataset's patient cohort.
Recruitment	There was no recruitment for this study.
Ethics oversight	*Identify the organization(s) that approved the study protocol.*

Note that full information on the approval of the study protocol must also be provided in the manuscript.

Field-specific reporting

Please select the one below that is the best fit for your research. If you are not sure, read the appropriate sections before making your selection.

☒ Life sciences ☐ Behavioural & social sciences ☐ Ecological, evolutionary & environmental sciences

For a reference copy of the document with all sections, see nature.com/documents/nr-reporting-summary-flat.pdf

Life sciences study design

All studies must disclose on these points even when the disclosure is negative.

Sample size	The investigated datasets were chosen for their large sample size and the diversity of data modalities they cover. The seven investigated datasets cover various types of medical images, electrocardiograms and tabular electronic health record data and thus allow drawing generalisable conclusions about medical AI privacy risks.
Data exclusions	The following pre-established exclusion criteria were used. For FairVision, N=1,458 images were excluded due to their poor quality, whereby images were filtered automatically based on simple heuristics (image-wise mean and s.d.). To prevent shortcut learning, we excluded N=120,164 mammograms from EMBED that were acquired in diagnostic (non-screening) settings or showed views other than craniocaudal or mediolateral oblique. No other exclusions were performed.
Replication	Record- and patient-level membership inference attack sucess rates where measured across a large (N=200) set of target models. For each target record, these models were evenly split such that N=100 contained the record in its training data while N=100 did not. All attempts at replication were successful.
Randomization	Target models were trained on random patient subsets and later partitioned by the inclusion/exclusion of each patient's data.
Blinding	All datasets used were anonymized.

Reporting for specific materials, systems and methods

We require information from authors about some types of materials, experimental systems and methods used in many studies. Here, indicate whether each material, system or method listed is relevant to your study. If you are not sure if a list item applies to your research, read the appropriate section before selecting a response.

Materials & experimental systems

n/a	Involved in the study	
☒	☐	Antibodies
☒	☐	Eukaryotic cell lines
☒	☐	Palaeontology and archaeology
☒	☐	Animals and other organisms
☒	☐	Clinical data
☒	☐	Dual use research of concern
☒	☐	Plants

Methods

n/a	Involved in the study	
☒	☐	ChIP-seq
☒	☐	Flow cytometry
☒	☐	MRI-based neuroimaging

Plants

Seed stocks	*Report on the source of all seed stocks or other plant material used. If applicable, state the seed stock centre and catalogue number. If plant specimens were collected from the field, describe the collection location, date and sampling procedures.*
Novel plant genotypes	*Describe the methods by which all novel plant genotypes were produced. This includes those generated by transgenic approaches, gene editing, chemical/radiation-based mutagenesis and hybridization. For transgenic lines, describe the transformation method, the number of independent lines analyzed and the generation upon which experiments were performed. For gene-edited lines, describe the editor used, the endogenous sequence targeted for editing, the targeting guide RNA sequence (if applicable) and how the editor was applied.*
Authentication	*Describe any authentication procedures for each seed stock used or novel genotype generated. Describe any experiments used to assess the effect of a mutation and, where applicable, how potential secondary effects (e.g. second site T-DNA insertions, mosiacism, off-target gene editing) were examined.*
