# Supplementary material for: Disparate privacy risks from medical AI
Source: Nature. 2026 Jun 24;656(8126):192–8. doi: 10.1038/s41586-026-10688-0 (PMC13442007; doi:10.1038/s41586-026-10688-0)
Supplement: Supplementary file 3 [file 41586_2026_10688_MOESM3_ESM.pdf]

nature portfolio

# Peer Review File

# Disparate privacy risks from medical AI

Corresponding Author: Mr Moritz Knolle

Any redactions in this file are there to maintain patient confidentiality, the confidentiality of unpublished data, or to remove third-party material.

**This file contains all reviewer reports in order by version, followed by all author rebuttals in order by version.**

Version 1:

Reviewer comments:

Referee #1

(Remarks to the Author)
The paper presents an examination of patient-level privacy in AI models trained for medical diagnosis. The paper builds on prior work showing that membership inference attack (MIA are an important hole in AI models, and find that MIA are very successful in individual patients, with higher attack success rate (ASR) in larger models, and in under-represented groups of patients. While not entirely novel (see citations below) this examination is warranted in a health setting with state-of-the-art AI models on a larger set of tasks and settings.

I would appreciate either more insight on the nature of the score/attacks in comparison to the existing MIA/disparate impact literature (see citations below), or more centred analysis on medical models and types of attacks that are relevant in the healthcare setting. As it stands the paper is not quite delivering on one of these, and the focal spread is difficult to make a strong contribution in.

https://iclr.cc/virtual/2024/poster/18131
https://proceedings.mlr.press/v139/choquette-choo21a.html
https://petsymposium.org/popets/2022/popets-2022-0023.pdf
https://dl.acm.org/doi/10.1145/3488932.3501279

Main Strengths
-----***-----
- The paper addresses an important gap in the wider literature - shifting from aggregate metrics to evaluating privacy risks at the individual level, which is especially relevant in healthcare.

- The authors includes a comprehensive suite of benchmarks and experiments, demonstrating thoughtful evaluation across various settings.

Major Weaknesses
-----***-----
- The link between MIA success and actual privacy risk is not clearly established. If the adversary already has access to the data, what additional risk does membership inference pose? Again, see "Can LLMs Keep a Secret? Testing Privacy Implications of Language Models via Contextual Integrity Theory" for an examination of privacy in context. Specifically, is the issue that models reveal private information, or that models reveal private information in contexts that humans would not?

- The paper is focusing on MIA for medical imaging models only - which lacks a clear argument for harm, e.g., in leaking someone's chest x-ray because that cannot be used to reconstruct identity or otherwise identify someone. For example head MRI example is a different case where we can indeed reconstruct from chest x-rays. Even then I think MIAs are a privacy concern that needs to be justified for healthcare models vs. reconstruction attacks. The more concerning result would be if it held for EHR models.

- The notion of "minority" is unclear here. Both figures and descriptions lack detail on how subgroups are defined, their size, and more description about the data distribution within these subgroups in comparison to the entire dataset. This specific

result - that MIAs are more successful for outliers / minority groups - has been shown in other papers, and might be expected, so more analysis is needed.

- Is there a reason the authors did not subsequently train these models with DP and evaluate the benefit regarding MIAs? Obviously this would improve the leakage, but I am curious if they can find a sweet spot (e.g., with a weak epsilon but good MIA performance). DP is reasonably low hanging fruit for these models, and it would solidfy our understanding to know how addressable these issues are.

- The proposed extension to LR-MIA seems computationally intensive, requiring training of many models. Scalability concerns, particularly on large datasets, are not sufficiently discussed here to warrant understanding of the extensibility.

- It is important to know the sensitivity of the score to the number of trained models required. What is a reasonable way for picking N? I see no ablations done here to confirm it, but this is important for usage in practice.

- How does the proposed method perform compared to existing MIA, for instance in comparison to a calibrated LR-MIA prediction?

Minor Points
-----***-----
- The terms target model and shadow model appear to be used interchangeably? My understanding is that the models that are trained to replicate the target model behaviour are called shadow model. Can you clarify why you mention “training target models”?

- Citation error: Page 4, first line contains an incorrect citation for LR-MIA.

- The paper shows that outliers are more vulnerable to MIA, but it’s unclear how inference models treat outlier samples not present in the training set. I’m curious to know if such samples are easier to identify or are systematically given higher probability of being part of the training data?

- A similar question arises for minority groups—is the inference model simply worse at predicting membership for them due to systematic model bias?

- The discussion says “efforts to collect diverse training datasets, which are urgently needed to build unbiased AI models which offer equitable performance on diverse patient populations, will require improving data- sharing trust relationships with minority groups” While this can be very true, I didn’t see anywhere in the paper showing the impact of bias in the data with inference attack. If there is such finding, it would be very interesting to highlight it.

(Remarks on code availability)

Referee #2

(Remarks to the Author)
This manuscript presents a systematic study of disparate privacy risks in medical AI, focusing on how membership inference attacks (MIAs) affect demographic subgroups differently. Using a variety of clinical datasets (including MIMIC-IV and MedMNIST) and machine learning models (neural networks, LightGBM, logistic regression), the authors find that individuals from underrepresented or marginalized demographic groups (e.g., racial minorities, the elderly, and people with rare health conditions) often face higher privacy risks in AI models. Importantly, these disparities persist even when group-level membership inference risks appear similar, highlighting a previously underappreciated layer of privacy inequity in clinical ML.

Originality and Significance:
The work is original and highly significant. While membership inference attacks have been studied in general contexts, the exploration of demographic disparities in privacy vulnerability is novel and timely. The paper offers a critical perspective on AI fairness by linking data protection and equity concerns. It extends and complements recent literature on fairness and privacy in ML, but focuses uniquely on subgroup-differentiated privacy vulnerabilities in healthcare, which has not been comprehensively explored. The findings are consequential for policy and practice, especially in regulated domains like healthcare where both fairness and privacy are essential.

Data and Methodology:
The study uses publicly available, well-established datasets (MIMIC-IV, MedMNIST, UTHealth Claims) with sufficient breadth and clinical realism to support generalizable claims. The methodology is robust: the use of multiple models, both black-box and white-box MIAs, and extensive subgroup analyses lend credibility to the results. The authors clearly define the statistical treatment of subgroup disparities (e.g., per-individual vs. aggregate privacy risk) and make a compelling case for disaggregating privacy risk evaluations. The presentation of the methods is generally strong, though some details (e.g., model hyperparameters, training epochs, and specific attack model structures) could be clarified in an appendix or supplementary material for reproducibility.

Statistical Considerations:

The statistical approach is appropriate for the questions posed. The authors correctly distinguish between individual-level and aggregate-level privacy risks, and quantify disparities using both absolute and relative comparisons. Uncertainty is reasonably addressed using confidence intervals and significance testing for key results. However, more formal statistical testing for between-group differences (e.g., bootstrapped CIs or adjusted p-values for multiple comparisons across subgroups) would further strengthen claims of disparity.

Conclusions:
The conclusions are supported by the data and analyses. The key claims—that (1) membership inference risks are not evenly distributed across demographic groups and (2) underrepresented groups tend to bear greater risks—are robust across datasets, models, and attack types. The implications for healthcare AI governance, particularly regarding data sharing, regulatory oversight, and ethical model deployment, are well grounded. Nonetheless, some claims (e.g., regarding the ethical obligation to assess subgroup privacy risk) could be more explicitly supported by normative frameworks or legal precedent (e.g., GDPR's fairness and data protection principles).

Suggested Improvements:
- Consider evaluating mitigation techniques (e.g., differential privacy, adversarial training) across subgroups to explore whether disparities persist even when using common defenses.
- Include subgroup sizes and baseline outcome distributions in each dataset to contextualize disparity results.
- Include an appendix with detailed hyperparameters and training configurations for reproducibility.
- Strengthen the conclusion with more direct recommendations for regulators, IRBs, and clinical ML practitioners.

References:
The manuscript is well-cited and builds on prior work in privacy attacks, machine learning fairness, and clinical data ethics. It references foundational work and more recent applications in biomedical ML. Additional citation of works that bridge differential privacy and health equity could provide more context to the work.

Clarity and Context:
The manuscript is well written and accessible to a broad audience in medical AI, privacy, and ethics. The abstract clearly captures the motivation, methodology, and findings. The introduction is compelling and contextualizes the problem well within the healthcare domain. The conclusions are appropriately cautious and forward-looking. Some sections could benefit from more detail, but overall the manuscript is clear, logical, and well-structured.

(Remarks on code availability)
The med_leak GitHub repository provides a well-structured and reproducible codebase for analyzing subgroup-specific privacy risks in medical AI models, as presented in this paper. It includes scripts for training models, executing membership inference attacks, and generating visualizations across multiple clinical datasets. The code leverages JAX for high-performance computation and is organized into modular components, facilitating adaptation to other datasets or model architectures. With clear documentation, this repository is a valuable resource for researchers and practitioners interested in privacy auditing and fairness in healthcare machine learning, and most importantly, replicating the experiments described in this submission.

Referee #3

(Remarks to the Author)
I co-reviewed this manuscript with one of the reviewers who provided the listed reports.

(Remarks on code availability)

Version 3:

Reviewer comments:

Referee #1

(Remarks to the Author)
Thank you to the authors for the many clarifications in the updated manuscript and response. These updates have improved the manuscript. In particular, the additional description of the minority groups, and the ablation on N is very informative.

One of my major remaining concerns, which was unaddressed in the response, is that several strands of prior work already document that membership-inference risk is heterogeneous. For example, disparate vulnerability across subpopulations has been analyzed with statistical tests and mitigation studies (e.g., Disparate Vulnerability to Membership Inference Attacks, https://arxiv.org/pdf/1906.00389); class-dependent variability in attack success has been measured at the label level (https://ieeexplore.ieee.org/document/9230385 ); and individual-level susceptibility was observed in early MIA formulations ( https://arxiv.org/pdf/1610.05820). For a paper of this magnitude, it is crucial that you make explicit how your LR-MIA setup, metrics, and clinical use case relate to and extend these lines, and highlight where your choices align with or differ from prior findings.

Below are the follow-up questions I have in response to the updated work, noted per comment:

--------------
Original comment: The link between MIA success and actual privacy risk is not clearly established. If the adversary already has access to the data, what additional risk does membership inference pose? Again, see "Can LLMs Keep a Secret? Testing Privacy Implications of Language Models via Contextual Integrity Theory" for an examination of privacy in context. Specifically, is the issue that models reveal private information, or that models reveal private information in contexts that humans would not?

Updated: The immunotherapy example is constructed narrowly so as to be strong and make your point well; it also highlights that privacy risk is nuanced and depends on many factors, for instance the population the model was trained on and related dataset/model characteristics. The text should clarify this nuance explicitly in the Introduction and revisit it in the Discussion so the manuscript accurately reflects how MI success rates translate into patient privacy risk across different settings, rather than raising a uniform concern.

--
Original comment: The paper is focusing on MIA for medical imaging models only - which lacks a clear argument for harm, e.g., in leaking someone's chest x-ray because that cannot be used to reconstruct identity or otherwise identify someone. For example, a head MRI example is a different case where we can indeed reconstruct from chest x-rays. Even then I think MIAs are a privacy concern that needs to be justified for healthcare models vs. reconstruction attacks. The more concerning result would be if it held for EHR models.

Updated: The new results on MIMIC and PTB-XL could substantially strengthen the paper, but require clarity on how the EHR attacks were conducted. Specifically, do success rates reflect attacks using the full patient record or partial records/subsets of features and time windows? This distinction matters enormously for interpreting privacy risk: successful membership inference given complete records implies a different (extremely low) real-world risk than success when only partial records are available.

--
Original comment: How does the proposed method perform compared to existing MIA, for instance in comparison to a calibrated LR-MIA prediction?

Updated: I would like to clarify the original question. By this question I meant taking the per-record LR (LiRA/RMIA) scores (optionally calibrated to membership probabilities, but this is not strictly required) and then examining the extreme tail of those scores to see who is most at risk. Could this potentially characterize those extremes to be latent subpopulations? This matters because, for instance, a patient with a very atypical diagnosis may not fall into any predefined subgroup yet still sit in the extreme-risk tail; clustering or describing features of the tail (e.g., diagnosis codes, test panels, site/device attributes) would reveal such cases.

--
Original comment: The paper shows that outliers are more vulnerable to MIA, but it's unclear how inference models treat outlier samples not present in the training set….. A similar question arises for minority groups.Is the inference model simply worse at predicting membership for them due to systematic model bias?

Updated: Regarding my concern about attack performance and its relation to data distribution, I'm clarifying my question. For example, a common driver of subgroup vulnerability is overfitting - where minority groups (with fewer samples or distinctive features) are often easier for the model to memorize. While you note efforts to prevent overfitting ("we undertake every effort…countermeasures to prevent overfitting"), the analysis doesn't disentangle how much of the observed MIA success reflects intrinsic vulnerability versus data effects like overfitting. To clarify the root cause, you can for example report subgroup-wise generalization metrics (test accuracy/loss), and member vs non-member loss distributions, and compare them to overall performance. Overall, brief correlation analysis between subgroup test error (or calibration) and LR-MIA power would make the causal story more convincing.

--
Original comment: The discussion says "efforts to collect diverse training datasets, which are urgently needed to build unbiased AI models which offer equitable performance on diverse patient populations, will require improving data- sharing trust relationships with minority groups" While this can be very true, I didn't see anywhere in the paper showing the impact of bias in the data with inference attack. If there is such finding, it would be very interesting to highlight it.

Updated: I agree that in practice the assessment of AI performance bias depends on choosing a fairness definition that is appropriate and relevant to the specific clinical setting, because this is application-specific. However, the authors can simply choose a plausible single - or set of - applications and demonstrate the impact of bias with inference attack in that setting? If the authors would rather remove all mentions of

(Remarks on code availability)

Referee #2

(Remarks to the Author)
This manuscript presents the first patient-level privacy audit of medical AI models, quantifying the risk that individual patients face from membership inference attacks (MIAs). Using seven medical datasets spanning imaging and non-imaging modalities (CheXpert, MIMIC-CXR, Fitzpatrick 17k, FairVision, EMBED, PTB-XL, and MIMIC-IV-ED), the authors demonstrate that MIAs can achieve near-perfect success rates for certain individuals and that the most vulnerable patients are disproportionately from underrepresented groups. Importantly, the work shows that larger, more diagnostically accurate models tend to amplify these vulnerabilities. The authors also validate differential privacy (DP) as an effective. but computationally demanding. mitigation strategy, finding that patient-level DP accounting may be necessary for full protection .

The study advances prior work by moving beyond aggregate privacy risk metrics to quantify individual-level vulnerability, an important conceptual and methodological step forward in AI privacy auditing. While prior studies examined record-level privacy leakage, this work applies those methods in a clinical context and connects privacy risk to health equity concerns. The authors' addition of non-imaging data (PTB-XL, MIMIC-IV-ED) and their focus on demographic disparities enhance both novelty and relevance. Their revisions explicitly contextualize findings within existing literature on health inequalities and AI fairness, which strengthens the paper's contribution to the state of knowledge.
The methodology is robust, well-motivated, and clearly described. The use of 200 independently trained target models per dataset provides statistically stable estimates of record- and patient-level MIA success, and the authors' ablation studies convincingly address scalability concerns raised in the prior review. The inclusion of two additional datasets (PTB-XL and MIMIC-IV-ED) directly addresses earlier critiques regarding generalizability beyond imaging models. The response document also clarifies terminology (target vs. shadow models) and strengthens statistical rigor through standard error estimation and explicit model performance benchmarking. The authors' revised results tables and expanded methodological detail enhance reproducibility and clarity.

The statistical analyses are appropriate and carefully executed. ROC-based AUC calculations for individual records and aggregated patient-level metrics are well justified, and the inclusion of 95% confidence intervals demonstrates proper uncertainty quantification. The authors' new ablation analysis on the number of target models (N) adds practical insight into the stability of MIA success estimates, a key concern for computational feasibility. Their treatment of subgroup comparisons using $\chi^2$ tests with Bonferroni correction and Pearson residuals for interpreting effect sizes is sound and appropriately conservative.

The conclusions are robust and consistent with the presented data. The manuscript persuasively argues that aggregate privacy metrics underestimate individual risk, that underrepresented groups face disproportionate exposure, and that privacy risk scales with model capacity. The finding that record-level differential privacy is insufficient for multi-record patients is particularly impactful and has clear policy and implementation implications. Overall, the conclusions are well supported, balanced, and framed in relation to both technical and ethical dimensions of medical AI deployment.

This revised manuscript represents a substantial improvement and now stands as a rigorous, methodologically sound, and ethically relevant contribution to the field of medical AI privacy. The authors have effectively addressed prior critiques, expanded their empirical base, and clarified theoretical implications. The paper makes a meaningful and timely contribution to understanding individual-level privacy risks in AI systems and offers actionable insights for implementing differential privacy in clinical AI pipelines.

(Remarks on code availability)
The Leakoscope repository provides a well-structured, complete, and reproducible implementation of patient-level privacy audits for medical AI models. The code is organized into modular components for data processing, model training, and privacy attack analysis, with clear directory separation and automation scripts that enable full replication of the published results. Comprehensive configuration options, dataset-specific preprocessing notebooks, and end-to-end execution scripts (e.g., train_all.sh, plot_all.sh) demonstrate a mature and reproducible workflow, although reproducing the complete experiments requires substantial computational resources. The inclusion of both Likelihood Ratio (LiRA) and Robust Membership Inference (RMIA) attack implementations, alongside integrated differential privacy experiments, indicates a high level of technical completeness and alignment with the methods described in the paper. Overall, the codebase appears to be of high research quality, suitable for reuse, adaptation, and extension by other investigators in AI privacy and medical data security.

Referee #3

(Remarks to the Author)
I co-reviewed this manuscript with one of the reviewers who provided the listed reports.

(Remarks on code availability)

Version 4:

Reviewer comments:

Referee #1

(Remarks to the Author)
I have no further major concerns, but I still think the tone is overly aggressive about risks without some context for practical risk (see prior reviews). I would appreciate this being addressed in the final version.

(Remarks on code availability)

Referee #3

(Remarks to the Author)
I co-reviewed this manuscript with one of the reviewers who provided the listed reports.

(Remarks on code availability)

**Open Access** This Peer Review File is licensed under a Creative Commons Attribution 4.0 International License, which permits use, sharing, adaptation, distribution and reproduction in any medium or format, as long as you give appropriate credit to the original author(s) and the source, provide a link to the Creative Commons license, and indicate if changes were made.
In cases where reviewers are anonymous, credit should be given to 'Anonymous Referee' and the source.
The images or other third party material in this Peer Review File are included in the article's Creative Commons license, unless indicated otherwise in a credit line to the material. If material is not included in the article's Creative Commons license and your intended use is not permitted by statutory regulation or exceeds the permitted use, you will need to obtain permission directly from the copyright holder.
To view a copy of this license, visit https://creativecommons.org/licenses/by/4.0/

# Point-by-point response to referee comments: 2025-01-00537A

To begin, we would like to sincerely thank the referees for their detailed and constructive feedback, which we think has substantially strengthened our work. We have revised the manuscript extensively according to their input and are confident that their concerns have been satisfactorily addressed. Please find our point-by-point response to their comments on the next page.

**Referee #1 (Remarks to the Author):**

> *… (summary and strengths section of the comments omitted for brevity)...*
>
> *Major Weaknesses*
> *-----***-----*
> *-" The link between MIA success and actual privacy risk is not clearly established. If the adversary already has access to the data, what additional risk does membership inference pose? Again, see "Can LLMs Keep a Secret? Testing Privacy Implications of Language Models via Contextual Integrity Theory" for an examination of privacy in context. Specifically, is the issue that models reveal private information, or that models reveal private information in contexts that humans would not?"*

The referee is correct that, in the unrealistic scenario where an attacker has complete knowledge of a patient's data, a MIA poses no actual privacy risk. However, in many practical scenarios, a successful MIA can reveal additional sensitive information about the patient that was not available to the attacker before. Consider the following example of a fictitious attack against a real-world model from *Yoo et al.* [1]:

> **Scenario:** A research centre trains an AI model that predicts the efficacy of immunotherapy for cancer patients using routinely collected blood test data [1]. An attacker gains access to a patient's routine blood test results and successfully performs a MIA on this model. This reveals that the patient has cancer, sensitive information that the attacker could not have obtained from the blood test results alone.

It is easy to imagine similar scenarios where a successful MIA could reveal: a mental health condition, a sexually transmitted disease, or a dormant genetic condition. Less severe violations are also possible. For many training dataset collection scenarios, a successful MIA can reveal to an attacker that the patient visited a specific centre during a specific time period. This information could be used to break anonymity through triangulation, i.e., inferring the patient's identity in another anonymised dataset collected at the same institution. Generally, however, it is evident that such a privacy violation can only occur in scenarios where the context in which the training dataset was collected is specific enough to allow drawing privacy-critical conclusions. As a result, the privacy risk posed by a MIA, in itself, is context-dependent and does not hold generally in all model deployment settings.

A more broadly applicable risk posed by MIAs stems from their usage as an integral component in modern data reconstruction attacks against generative models [2-4]. These attacks allow for the extraction of individual records from a model's training dataset and thus pose a severe privacy risk to data contributors. Crucially, the privacy risk posed by these attacks is universal, regardless of deployment or dataset scenario. Modern data reconstruction attacks follow a simple two-step procedure:

1.) Generate a large quantity of data using the generative model (either through some prompting strategy or with an empty prompt),

2.) Filter through this data with a MIA and identify records that were part of the training dataset.

Prior research has shown that this simple procedure, which does not require access to any target data, is extremely effective at extracting individual training data records from image-based diffusion models [2] (e.g., Stable Diffusion), large language models (LLMs) [3] and more recently, commercial LLMs [4] (e.g., OpenAI's GPT models). Viewed in the context of these studies, our key finding that MIAs are exceptionally effective for individual patients is highly concerning because it has far-reaching consequences for individuals whose data is used to train large generative models, as is standard practice in industry. We have modified the introduction and discussion in the revised manuscript to clarify: (1) how MIAs can cause privacy violations and (2) the connection between MIAs and modern reconstruction attacks.

The modified section in the introduction now reads:

*"For example, a membership inference attack (MIA) attempts to determine whether a specific patient's data was part of a model's training dataset. In some scenarios, this can constitute a privacy violation in itself, e.g., a successful MIA against a model that predicts immunotherapy efficacy from routine blood test data [1] reveals that the patient has cancer. Notably, the privacy risk posed by MIAs is not limited to specific scenarios. A more broadly applicable risk from MIAs stems from their usage in modern data reconstruction attacks against generative models [2, 3, 4] where they are used as oracles to verify that extracted records were part of a model's training dataset."*

The modified section in the discussion now reads:

*"Our key finding that MIAs against AI models are exceptionally effective for individual data contributors has important implications. MIAs play a crucial role in modern data reconstruction attacks on generative AI models, where they are used to filter through large amounts of generated candidate records. Reconstruction attacks allow for the extraction of individual records from a model's training dataset and have been demonstrated for diffusion-based image generation models [2], large language models [3], and recently, aligned, production-level large language models [4]. While our study focused on discriminative (diagnostic) AI models, the type of attack we studied (LR-MIA) is generally applicable and can be used against generative models without major modifications. We thus see the exploration of our proposed methodology for estimating record- and patient-level MIA success against generative models as an interesting direction for future research. Given the substantial computational resources this would require, exploring scalable approximation techniques is another valuable avenue to investigate."*

The second part of the referee's comment (highlighted in yellow) references a paper which studies privacy risks associated with providing sensitive data to a generative model at inference/deployment time, i.e., situations where a user provides sensitive information to an LLM chatbot. As this paper does not focus on privacy aspects of the training data, but rather on additional data provided at deployment time, it is not relevant to our submission.

> *"- The paper is focusing on MIA for medical imaging models only - which lacks a clear argument for harm, e.g., in leaking someone's chest x-ray because that cannot be used to reconstruct identity or otherwise identify someone. For example head MRI example is a different case where we can indeed reconstruct from chest x-rays. Even then I think MIAs are a privacy concern that needs to be justified for healthcare models vs. reconstruction attacks. The more concerning result would be if it held for EHR models."*

To directly address this referee's concern regarding the limited generalisability of our findings, we have conducted additional experiments on two non-imaging datasets: PTB-XL [5] (electrocardiograms, N=18,869 patients) and MIMIC-IV-ED [6] (electronic health records, N=201,213 patients). Experimental results from these datasets demonstrate that our key findings on medical imaging datasets generalise directly to other modalities, notably including EHR models, as questioned by the referee. We have integrated this additional data into the revised manuscript (see Fig. 2b & c); subgroup comparisons for these new datasets are shown in Fig. 3d, & Fig. A2c. In brief, these data corroborate our key finding that MIA success rates are exceptionally high for a small subset of patients, while aggregate attack success does not indicate this vulnerability.

Finally, we would like to remark that there is strong evidence contradicting the referee's statement that chest radiographs cannot be used to re-identify patients. For one, *Packhäuser et al.* [7] showed that patients can be re-identified from chest radiographs with remarkably high success rates using simple AI techniques (siamese networks). Moreover, there is a general consensus in the privacy research community that the protection offered by anonymisation is generally imperfect: the re-identification of individuals is not limited to specific data modalities but is generally possible with any kind of anonymised data. *Rocher et al.* [8] recently proposed a general scaling law to model the effectiveness of re-identification techniques and showed that such techniques can scale to re-identify individuals in comparison pools comprising data from billions of individuals.

> *"- The notion of "minority" is unclear here. Both figures and descriptions lack detail on how subgroups are defined, their size, and more description about the data distribution within these subgroups in comparison to the entire dataset. This specific result - that MIAs are more successful for outliers/minority groups - has been shown in other papers, and might be expected, so more analysis is needed."*

We thank the referee for highlighting the lack of clarity and agree that the term "minority", due to its additional meaning from the social sciences, is problematic and could lead to confusion. To address this issue, we have replaced all relevant mentions of "minority" with more precise terminology (smallest group or under-represented group) to make clear that we are referring to the relative size of a group in a dataset only (as measured by the number of contributed records). Furthermore, we have modified figure captions to include information on subgroup stratification and additionally added tables summarising all available information (e.g., age, sex, disease prevalence, etc.) about the patient cohorts, as well as relevant subgroups (Tables A1 - A7). We would also like to point out that the relative size of each group is indicated in all figures showing data from subgroup comparisons (in Fig. 3 & A2 by bar colour and in Fig. A3 on the x-axis).

At this point, we would like to differentiate our work from existing studies mentioned by the referee, which have shown that MIA success rates differ between subgroups. These studies report differences in aggregate MIA success between subgroups on small, low-dimensional datasets using shallow neural network models, and their findings thus offer limited generalizability for two reasons. First, prior work by *Carlini et al.* [9] showed that findings from MIAs against shallow neural networks trained on low-dimensional datasets generalise poorly to high-dimensional datasets and modern (deep neural network) AI models. Second, one of our key findings is that aggregate MIA success is an unsuitable measure of privacy risk, as the metric is dominated by the large number of records for which the attack only reaches random-guessing performance. Thus, as a direct consequence of this finding, subgroup

differences in aggregate attack success also offer limited insight into privacy risk differences between groups.
In contrast to the existing works mentioned by the referee, our study moves beyond subgroup differences in aggregate MIA success and investigates subgroup composition differences in the subset of records most susceptible to MIAs (99th record-level MIA AUC percentile) compared to the overall dataset. In other words, we measure how often, relatively speaking, a given subgroup appears among the records most susceptible to a MIA and compare this to the relative number of records the subgroup contributes to the overall dataset. Thus, while the conclusions of our study and the existing literature appear similar at first glance, the quantification of risk differences between groups is fundamentally different.

> *"- Is there a reason the authors did not subsequently train these models with DP and evaluate the benefit regarding MIAs? Obviously this would improve the leakage, but I am curious if they can find a sweet spot (e.g., with a weak epsilon but good MIA performance). DP is reasonably low hanging fruit for these models, and it would solidify our understanding to know how addressable these issues are."*

We did not conduct these experiments for the original submission due to their high computational costs. However, we fully agree with the referee that investigating AI models protected by differential privacy (DP) and, more importantly, the relationship between the level of privacy protection (e.g., as measured by $\epsilon$ in $(\epsilon, \delta)$-DP) and patient-level attack success rates is of high interest to practitioners or policy makers. To directly address this concern, we conducted **extensive additional experiments** on AI models with various levels of record-level $(\epsilon, \delta)$-DP privacy protection for two datasets: EMBED (imaging) and PTB-XL (non-imaging). Data from these experiments have been integrated into the revised manuscript (see Fig. 2d & e) and suggest that, as expected, MIA success rates decrease for all patients with increasing levels of record-level DP protection. However, interestingly, we also found that the full mitigation of MIAs for all data-contributing patients requires stricter levels of privacy protection (smaller $(\epsilon, \delta)$) than previously believed [9,11]. Moreover, our results also show that fully mitigating MIAs requires DP accounting at the patient- rather than the record-level. This suggests that record-level DP protection, which is commonly used for its simplicity, is not sufficient to completely prevent privacy attacks on an AI model that is trained on a dataset where one individual contributes multiple records.

> *"- The proposed extension to LR-MIA seems computationally intensive, requiring training of many models. Scalability concerns, particularly on large datasets, are not sufficiently discussed here to warrant understanding of the extensibility."*

We would like to clarify that we do not propose an extension to LR-MIA. Instead, we propose a simple and efficient technique to estimate the success rate of LR-MIAs at record/patient-level resolution. Estimating MIA success rates at patient-level resolution, by definition, requires measuring attack success rates -independently for each patient's records- across many target models (see Fig. 1c). Conducting a privacy audit at this level of granularity is thus necessarily a computationally expensive endeavour. Notably, the only other work that measures attack success at record-level granularity uses N=20,000 target models [12]. Besides the order of two magnitudes efficiency gain (20,000 vs. 200), our method has two advantages: (1) it requires target models only, and (2) it provides standard error estimates for each patient's attack success rate. In a practical setting, an auditor can thus easily gauge the standard error of each

patient's MIA success estimate and adaptively decide whether more computational resources should be allocated to reduce the estimation error further (by training more target models) or whether the current level of error is satisfactory for their use case. See our response directly below for our assessment of an extensive ablation study on computational resources and their impact on the standard error of our record-level MIA success estimates.

> *"- It is important to know the sensitivity of the score to the number of trained models required. What is a reasonable way for picking N? I see no ablations done here to confirm it, but this is important for usage in practice."*

This is an excellent question that is highly relevant to practitioners. We have conducted an extensive ablation study on $N$ (the number of target models used for record-level MIA success estimation) across all investigated datasets. Data from this ablation study (see Fig. A4) suggests that the standard error for all record-level MIA AUC scores decreases quickly with an increasing number of target models. Crucially, the standard error among the most vulnerable records (99th record-level MIA AUC percentile) decreases even faster. This effect is particularly pronounced for PTB-XL and Fitzpatrick 17k, the two datasets with the highest MIA vulnerability. This property –that the standard error drops rapidly for the 99th percentile of vulnerable records– is useful for practitioners as it allows identifying the most vulnerable records/patients with modest computational resources.

> *"- How does the proposed method perform compared to existing MIA, for instance in comparison to a calibrated LR-MIA prediction?"*

Could the referee please clarify what they mean by calibrated LR-MIA prediction? *LiRA* [9] and *RMIA* [13], the state-of-the-art MIAs we investigate in our study, are difficulty-calibrated to every target record. In essence, these attacks exploit relative changes in confidence when a record is included in/excluded from a model's training dataset. Both of these attacks (LiRA and RMIA) are constructed using a likelihood-ratio test on model confidence values and are thus likely quite close to the theoretically-optimal performance of a statistical test for membership inference (see Neyman-Pearson Lemma [14]). Since LiRA and RMIA are widely considered the most powerful existing attacks, we are unsure whether comparisons to other MIAs would yield any further insights.

> *"Minor Points*
> *-----***-----*
> *- The terms target model and shadow model appear to be used interchangeably? My understanding is that the models that are trained to replicate the target model behaviour are called shadow model. Can you clarify why you mention "training target models"?"*

We'd like to clarify this point regarding the use of "target model" and "reference model" (also referred to as "shadow model" in the wider literature). These terms have been used with great care and rigour throughout the entire manuscript to denote: models used to compute statistics needed to conduct LR-MIAs, and models used to evaluate MIA success, respectively (analogous to training and testing data in classical machine learning).

To measure MIA success, one must know the ground truth membership status, i.e., which records were actually included or excluded from the target model's training data. As is standard

practice –in virtually all publications on MIAs– attack success is measured by 'training a target model' on a random subset of the training dataset, then evaluating the attack's success in aggregate across all records (as illustrated in Fig. 1a). Our study follows this established methodology but crucially measures MIA success at a finer, record/patient-level granularity. This requires evaluating attack success independently for each target record across many (N=200) target models (as shown in Fig. 1c). Note that these target models are split evenly such that for every target record, there are N=100 target models that included the record in their training dataset and N=100 target models that did not. For a more detailed explanation of our process for measuring record/patient-level MIA success, please refer to the section titled "From aggregate to patient-level privacy risk estimation."

> *"- Citation error: Page 4, first line contains an incorrect citation for LR-MIA."*

Thank you for spotting this error; we have corrected it in the revised manuscript.

> *"- The paper shows that outliers are more vulnerable to MIA, but it's unclear how inference models treat outlier samples not present in the training set. I'm curious to know if such samples are easier to identify or are systematically given higher probability of being part of the training data?"*

The referee's concern that there is a lack of clarity around how LR-MIAs treat outlier samples that were not part of the training dataset is unfounded, as this point has been addressed in prior work by *Carlini et al.* [9]. The key innovation behind LR-MIAs, and the reason why this type of attack is so powerful, is that the attack is independently calibrated for each and every target record. In essence, LR-MIAs compare, for each target record, the relative change in confidence when the record is included in/excluded from a model's training dataset. This means that outliers, which were not part of the training dataset, are not assigned a higher probability of being part of the training data than any other 'normal' record. On the other hand, a large change in confidence is expected for outliers that were part of the training dataset; thus, naturally explaining why MIAs on such records are so effective. While it is true that earlier MIAs (such as, e.g., the loss threshold attack by *Yeom et al.* [15]) had a tendency for the behaviour on outlier records described by the referee, LR-MIAs do not suffer from this problem.

> *"- A similar question arises for minority groups—is the inference model simply worse at predicting membership for them due to systematic model bias?"*

We do not understand this question and would kindly ask the referee to clarify what exactly they mean.

> *"- The discussion says "efforts to collect diverse training datasets, which are urgently needed to build unbiased AI models which offer equitable performance on diverse patient populations, will require improving data- sharing trust relationships with minority groups" While this can be very true, I didn't see anywhere in the paper showing the impact of bias in the data with inference attack. If there is such finding, it would be very interesting to highlight it."*

While we share the referee's opinion that this is an interesting topic to investigate, the assessment of AI performance biases hinges on choosing a fairness definition that is

appropriate and relevant to the specific clinical setting in which a model is deployed. Picking a relevant fairness definition is thus application-specific and would likely yield different technical definitions/metrics for each of the datasets investigated in our study. As a result, we deem this request beyond the scope of the paper and have removed the relevant sentence from the discussion section to improve its clarity and avoid confusion.

**Referee #2 (Remarks to the Author):**

> *"This manuscript presents a systematic study of disparate privacy risks in medical AI, focusing on how membership inference attacks (MIAs) affect demographic subgroups differently. Using a variety of clinical datasets (including MIMIC-IV and MedMNIST) and machine learning models (neural networks, LightGBM, logistic regression), the authors find that individuals from underrepresented or marginalized demographic groups (e.g., racial minorities, the elderly, and people with rare health conditions) often face higher privacy risks in AI models. Importantly, these disparities persist even when group-level membership inference risks appear similar, highlighting a previously underappreciated layer of privacy inequity in clinical ML.*
>
> *Originality and Significance:*
> *The work is original and highly significant. While membership inference attacks have been studied in general contexts, the exploration of demographic disparities in privacy vulnerability is novel and timely. The paper offers a critical perspective on AI fairness by linking data protection and equity concerns. It extends and complements recent literature on fairness and privacy in ML, but focuses uniquely on subgroup-differentiated privacy vulnerabilities in healthcare, which has not been comprehensively explored. The findings are consequential for policy and practice, especially in regulated domains like healthcare where both fairness and privacy are essential.*
>
> *Data and Methodology:*
> *The study uses publicly available, well-established datasets (MIMIC-IV, MedMNIST, UTHealth Claims) with sufficient breadth and clinical realism to support generalizable claims. The methodology is robust: the use of multiple models, both black-box and white-box MIAs, and extensive subgroup analyses lend credibility to the results. The authors clearly define the statistical treatment of subgroup disparities (e.g., per-individual vs. aggregate privacy risk) and make a compelling case for disaggregating privacy risk evaluations."*

The above sections marked in yellow are factually incorrect statements about the datasets/models/methods used in our original submission; none of these datasets or techniques were used in our original submission.

> *"The presentation of the methods is generally strong, though some details (e.g., model hyperparameters, training epochs, and specific attack model structures) could be clarified in an appendix or supplementary material for reproducibility."*

All methodological details that the referee requests here are described in sufficient detail to reproduce our findings and can be found in the Methods section (see section "General model

training details” & Table 1). We would kindly ask the referee to point out exactly which additional details are missing, and we will add them.

> *“Statistical Considerations:*
> *The statistical approach is appropriate for the questions posed. The authors correctly distinguish between individual-level and aggregate-level privacy risks, and quantify disparities using both absolute and relative comparisons. Uncertainty is reasonably addressed using confidence intervals and significance testing for key results. However, more formal statistical testing for between-group differences (e.g., bootstrapped CIs or adjusted p-values for multiple comparisons across subgroups) would further strengthen claims of disparity.”*

We thank the referee for the suggested improvement of our methodology in order to ensure reliable statistical conclusions. To directly address this comment, we have corrected for multiple comparisons across all subgroup comparisons performed for a single dataset using the Bonferroni method (see Fig. 3 & A2 for the updated results).

> *“Conclusions:*
> *The conclusions are supported by the data and analyses. The key claims—that (1) membership inference risks are not evenly distributed across demographic groups and (2) underrepresented groups tend to bear greater risks—are robust across datasets, models, and attack types. The implications for healthcare AI governance, particularly regarding data sharing, regulatory oversight, and ethical model deployment, are well grounded. Nonetheless, some claims (e.g., regarding the ethical obligation to assess subgroup privacy risk) could be more explicitly supported by normative frameworks or legal precedent (e.g., GDPR’s fairness and data protection principles).”*

We fully agree with the referee on the importance of the ethical and legal implications of our findings. Our expertise, however, is concentrated on machine learning for medicine, and we've thus consciously refrained from commenting on the legislative implications of our work, such as for the GDPR. We believe it's crucial for legal experts to lead the interpretation of our findings in this domain. That said, we would be happy to provide explanations of the technical details to legal experts or policymakers to help guide the interpretation of our findings.

> *“Suggested Improvements:*
> *- Consider evaluating mitigation techniques (e.g., differential privacy, adversarial training) across subgroups to explore whether disparities persist even when using common defenses.*
> *- Include subgroup sizes and baseline outcome distributions in each dataset to contextualize disparity results.*
> *- Include an appendix with detailed hyperparameters and training configurations for reproducibility.*
> *- Strengthen the conclusion with more direct recommendations for regulators, IRBs, and clinical ML practitioners.”*
>
> *… (remaining positive comments omitted for brevity)...*

We have integrated all of the above-mentioned suggestions into the revised manuscript. Please see the following sections:

- Investigation of models protected by differential privacy (Fig. 2d & e)
- Subgroup sizes and detailed information about patient cohorts (see Table A1-A7)
- Detailed hyperparameters and training configurations (Table 1)
- More concrete recommendations for models protected by differential privacy (discussion section)

The relevant, updated section in the discussion now reads:

*“Our experimental data confirmed that stronger levels of DP protection effectively reduce MIA success for all data-contributing patients. However, interestingly, we also observed that mitigating MIAs requires stronger levels of DP protection than previously thought [9,11]. More specifically, our results indicate that fully mitigating MIAs for all patients requires implementing DP protection at the patient- rather than the record-level.“*

**Additional General Remark:**
While this was not requested by any of the referees, we have made a minor technical improvement in the estimation procedure for patient-level MIA success. Our method requires training AI models on randomly sampled subsets of the training dataset. Before the modification, these subsets were sampled without taking into account which patient the included/excluded records belonged to. This led to an understimation of MIA success for patients who contribute many similar records. Our improved approach now samples subsets at the patient-level, thus ensuring that either all, or none, of a patient’s records are included to train a single model.

We are confident that this change from record- to patient-level sampling substantially improves the estimation of attack success for patients who contribute many similar records. Please note that this change has caused slight deviations in some of the experimental data compared to the original submission. All major trends and findings, however, remain consistent.

**Addressing the DURC Concerns:**

To begin, we would like to point out that our study does not introduce any novel methodology that could be used to cause privacy harm to individuals. The MIAs we investigate were proposed in [9, 13] and have been available as open-source software for several years now. Instead, our study proposes a method to granularly measure the success rates of this existing type of attack at patient-level resolution. This allows auditors to identify the most vulnerable records/patients in a dataset, a prerequisite for building AI systems that offer meaningful protection against privacy attacks.

Next, we would like to address the potential negative impacts that could arise from the widespread dissemination of knowledge that MIAs against AI models are remarkably effective for individual records/data contributors. This could lead to an increase in the number of attack attempts against medical AI models, which, if successful, can lead to the disclosure of sensitive patient information. While we acknowledge that this is a serious concern (particularly for large, generative models), we strongly believe that withholding knowledge of this vulnerability would have a much greater negative impact. Concurrent research by *Aerni et al.* [12] has presented similar record-level MIA success results to ours, albeit on natural image datasets, and thus it would only be a matter of time before other researchers arrive at the same conclusions as those presented in our work.

Overall, our work provides a positive outlook as we also demonstrate how the discovered privacy vulnerabilities can be mitigated. With the revised manuscript, we now present empirical evidence that increasing levels of record-level DP protection effectively reduces MIA success for all data-contributing patients. Crucially, however, our results also indicate that fully mitigating MIAs for all data-contributing patients requires DP accounting at the patient- rather than the record-level. This information could be valuable to policymakers as it indicates that record-level DP accounting, which is commonly used across academia and industry for its simplicity, is not sufficient to fully prevent privacy attacks against AI models trained on datasets where individual patients contribute multiple records.

Given the above points, we strongly believe that the benefit of making the results of our research publicly available outweighs any potential risks.

**References:**


[1] Yoo, Seong-Keun, et al. "Prediction of checkpoint inhibitor immunotherapy efficacy for cancer using routine blood tests and clinical data." *Nature Medicine* 31.3 (2025): 869-880.

[2] Carlini, Nicolas, et al. "Extracting training data from diffusion models." *32nd USENIX Security Symposium (USENIX Security 23)*. 2023.

[3] Carlini, Nicholas, et al. "Extracting training data from large language models." *30th USENIX Security Symposium (USENIX Security 21)*. 2021.

[4] Nasr, Milad, et al. "Scalable extraction of training data from aligned, production language models." *The Thirteenth International Conference on Learning Representations*. 2025.

[5] Wagner, Patrick, et al. "PTB-XL, a large publicly available electrocardiography dataset." *Scientific data* 7.1 (2020): 1-15.

[6] Xie, Feng, et al. "Benchmarking emergency department prediction models with machine learning and public electronic health records." *Scientific Data* 9.1 (2022): 658.

[7] Packhäuser, Kai, et al. "Deep learning-based patient re-identification is able to exploit the biometric nature of medical chest X-ray data." *Scientific Reports* 12.1 (2022): 14851.

[8] Rocher, Luc, Julien M. Hendrickx, and Yves-Alexandre de Montjoye. "A scaling law to model the effectiveness of identification techniques." *Nature Communications* 16.1 (2025): 347.

[9] Carlini, Nicholas, et al. "Membership inference attacks from first principles." *2022 IEEE symposium on security and privacy (SP)*. IEEE, 2022.

[10] Fawcett, Tom. "An introduction to ROC analysis." *Pattern recognition letters* 27.8 (2006): 861-874.

[11] Nasr, Milad, et al. "Adversary instantiation: Lower bounds for differentially private machine learning." *2021 IEEE Symposium on security and privacy (SP)*. IEEE, 2021.

[12] Aerni, Michael, Jie Zhang, and Florian Tramèr. "Evaluations of machine learning privacy defenses are misleading." *Proceedings of the 2024 on ACM SIGSAC Conference on Computer and Communications Security*. 2024.

[13] Zarifzadeh, Sajjad, Philippe Liu, and Reza Shokri. "Low-Cost High-Power Membership Inference Attacks." *International Conference on Machine Learning*. PMLR, 2024.

[14] Neyman, Jerzy, and Egon Sharpe Pearson. "On the problem of the most efficient tests of statistical hypotheses." *Philosophical Transactions of the Royal Society of London. Series A, Containing Papers of a Mathematical or Physical Character* 231.694-706 (1933): 289-337.

[15] Yeom, Samuel, et al. "Privacy risk in machine learning: Analyzing the connection to overfitting." *2018 IEEE 31st computer security foundations symposium (CSF)*. IEEE, 2018.

# Point-by-point response: 2025-01-00537B-Z

To begin, we would like to sincerely thank the referees for their detailed and constructive feedback, which we think has substantially strengthened our work. We have revised the manuscript extensively according to their input and are confident that their concerns have been satisfactorily addressed. Please find our point-by-point response to their comments on the next pages.

# Response to editorial comments:

## Contextualising our findings in the broader literature on health inequalities

> *"We ask that you delineate which datasets contain demographic data and how that data was collected. We also ask that additional efforts be made to contextualise the findings/ claims here with existing literature on health inequalities, and how medical AI in this context contributes to existing medical inequalities of minority individuals and groups."*

Our work reveals that the privacy risk burden which arises from the deployment of a medical AI model is not shared equally among data contributors. We present evidence that this disparity exists at two different levels:

**Individual-Level Disparity.** Our primary finding is that MIAs against AI models are exceptionally effective for some data-contributing patients, while for many others, the attack only reaches random-guessing performance. This suggests that some individuals are subject to high privacy risks because they provided consent that their personal data be used to train an AI model; others remain essentially unaffected. To the best of our knowledge, this aspect –that individuals may experience high (privacy) risk because they contributed their personal medical data to help others– is novel and has not been reported in the existing literature on health inequalities.

**Group-Level Disparity.** Our secondary finding is that there are significant differences in MIA success between patient subgroups. Strikingly, we found that these differences are driven, at least to some degree, by group size differences in the training data. Groups of patients that are underrepresented in a model training dataset are often over-represented among the records most susceptible to MIAs. By contrast, the opposite often holds true for majority groups. This aspect –that a disproportionately large share of the AI privacy risk burden rests on underrepresented (minority) groups– complements the existing literature on health inequalities [16], which has reported worse health outcomes and life expectancy for minority groups. Worryingly, our findings suggest that current trends in medical AI development and deployment could exacerbate these health inequalities. Prior research has shown that the diagnostic performance of AI models, which typically increases with the amount of suitable training data, can be significantly lower for underrepresented (minority) groups [17-19]. Thus, there is a possibility for a vicious cycle whereby minority groups place decreasing levels of trust in AI model performance and security, leading to a decreased willingness to contribute to model training datasets. To improve the diversity of training datasets and combat this vicious cycle, communication campaigns targeted towards minority groups will be needed.

We have modified the discussion section to reflect the above point on group-level disparities. The relevant section now reads:

*"We found significant differences in privacy risk between patient subgroups.The fact that some of these groups (e.g., race groups in chest radiographs) are not readily distinguishable by human experts raises concerns that privacy risk differences, which likely exist beyond the stratification variables we investigated, may pass unnoticed in practice. Strikingly, we found that the observed risk differences are driven, at least to some degree, by group size differences in*

*the training data. Groups of patients that are underrepresented in a model training dataset are often overrepresented among the records most susceptible to MIAs. By contrast, the opposite often holds true for majority groups. This finding –that a disproportionately large share of the AI privacy risk burden rests on underrepresented groups– complements the existing literature on health inequalities, which has reported worse health outcomes and life expectancy for marginalised/minority groups [16]. Worryingly, our findings suggest that current trends in medical AI development and deployment could exacerbate these health inequalities. Prior research has shown that the diagnostic performance of AI models, which typically increases with the amount of suitable training data, can be significantly lower for underrepresented (minority) groups [17-19]. Thus, we see a possibility for a vicious cycle whereby minority groups place decreasing levels of trust in AI model performance and security, leading to a decreased willingness to contribute to model training datasets. To improve the diversity of training datasets and combat this vicious cycle, communication efforts targeted towards minority groups will be needed."*

Finally, for each of the investigated datasets, we have added statements to the respective Methods subsection which details any available demographic information. Unfortunately, for many of the investigated datasets, information regarding the process by which the demographic data was collected is scarce. Nevertheless, we have added any information that we could find in the original publications to the respective Methods subsections.

# Response to referee comments:

## Referee #1 (Remarks to the Author):

*… (summary and strengths section of the comments omitted for brevity)...*

*“Major Weaknesses*
*-----***-----*
*- The link between MIA success and actual privacy risk is not clearly established. If the adversary already has access to the data, what additional risk does membership inference pose? Again, see "Can LLMs Keep a Secret? Testing Privacy Implications of Language Models via Contextual Integrity Theory" for an examination of privacy in context. Specifically, is the issue that models reveal private information, or that models reveal private information in contexts that humans would not?”*

The referee is correct that, in the unrealistic scenario where an attacker has complete knowledge of a patient’s data, a MIA poses no actual privacy risk. However, in many practical scenarios, a successful MIA can reveal additional, highly sensitive information about the patient that was not available to the attacker before. Consider the following example of a hypothetical, yet realistic attack against the real-world model from *Yoo et al.* [1]:

> **Scenario:** A research centre trains an AI model that predicts the efficacy of immunotherapy for cancer patients (with confirmed diagnosis) using routinely collected blood test data [1]. An attacker gains access to a patient’s routine blood test results and successfully performs a MIA on this model. This reveals that the patient has cancer, sensitive information that the attacker could not have obtained from the blood test results alone.

It is easy to imagine similar scenarios where a successful MIA could reveal: a mental health condition, a sexually transmitted disease, or a dormant genetic condition. Less severe violations are also possible. For many training dataset collection scenarios, a successful MIA can reveal to an attacker that the patient visited a specific centre during a specific time period. This information could be used to break anonymity through triangulation, i.e., inferring the patient’s identity in another anonymised dataset collected at the same institution. Generally, however, it is evident that such a privacy violation can only occur in scenarios where the context in which the training dataset was collected is specific enough to allow drawing privacy-critical conclusions. As a result, the privacy risk posed by a MIA, in itself, is context-dependent and does not hold generally in all model deployment settings.

A more broadly applicable risk posed by MIAs stems from their usage as an integral component in modern data reconstruction attacks against generative models [2-4]. These attacks enable the extraction of individual records from a model’s training dataset and thus pose a severe privacy risk to data contributors. Crucially, the privacy risk posed by these attacks is universal, regardless of deployment or dataset scenario. Modern data reconstruction attacks follow a simple two-step procedure:

1.) Generate a large quantity of data using the generative model (either through some prompting strategy or with an empty prompt),

2.) Filter through this data with a MIA and identify records that were part of the training dataset.

Prior research has shown that this simple procedure, which does not require access to any target data, is extremely effective at extracting individual training data records from image-based diffusion models [2] (e.g., Stable Diffusion), large language models (LLMs) [3] and more recently, commercial LLMs [4] (e.g., OpenAI's GPT models). Viewed in the context of these studies, our key finding that MIAs are exceptionally effective for individual patients is highly concerning because it has far-reaching consequences for individuals whose data is used to train large generative models, as is standard practice in industry. We have modified the introduction and discussion in the revised manuscript to clarify: (1) how MIAs can cause privacy violations and (2) the connection between MIAs and modern reconstruction attacks.

The modified section in the introduction now reads:

*"For example, a membership inference attack (MIA) attempts to determine whether a specific patient's data was part of a model's training dataset. In some scenarios, this can constitute a privacy violation in itself, e.g., a successful MIA against a model that predicts immunotherapy efficacy from routine blood test data [1] reveals that the patient has cancer. Notably, the privacy risk posed by MIAs is not limited to specific scenarios. A more broadly applicable risk from MIAs stems from their usage in modern data reconstruction attacks against generative models [2, 3, 4] where they are used as oracles to verify that extracted records were part of a model's training dataset."*

The modified section in the discussion now reads:

*"Our key finding that MIAs against AI models are exceptionally effective for individual data contributors has important implications. MIAs play a crucial role in modern data reconstruction attacks on generative AI models, where they are used to filter through large amounts of generated candidate records. Reconstruction attacks allow for the extraction of individual records from a model's training dataset and have been demonstrated for diffusion-based image generation models [2], large language models [3], and recently, aligned, production-level large language models [4]. While our study focused on discriminative (diagnostic) AI models, the type of attack we studied (LR-MIA) is generally applicable and can be used against generative models without major modifications. We thus see the exploration of our proposed methodology for estimating record- and patient-level MIA success against generative models as an interesting direction for future research. Given the substantial computational resources this would require, exploring scalable approximation techniques is another valuable avenue to investigate."*

Finally, the second part of the referee's comment references a paper which studies privacy risks associated with providing sensitive data to a generative model at inference/deployment time, i.e., situations where a user provides sensitive information to an LLM chatbot. As this paper does not focus on privacy aspects of the training data, but rather on additional data provided at deployment time, it is not relevant to our submission.

> *"- The paper is focusing on MIA for medical imaging models only - which lacks a clear argument for harm, e.g., in leaking someone's chest x-ray because that cannot be used to reconstruct identity or otherwise identify someone. For example head MRI example is a different case where we can indeed reconstruct from chest x-rays. Even then I think MIAs are a privacy concern that needs to be justified for healthcare models vs. reconstruction attacks. The more concerning result would be if it held for EHR models."*

To directly address the referee's concern regarding the limited generalisability of our findings, we have conducted additional experiments on two non-imaging datasets: PTB-XL [5] (electrocardiograms, N=18,869 patients) and MIMIC-IV-ED [6] (electronic health records, N=201,213 patients). Experimental results from these datasets demonstrate that our key findings on medical imaging datasets generalise directly to other modalities, notably including EHR models, as questioned by the referee. We have integrated this additional data into the revised manuscript (see Fig. 2b & c); subgroup comparisons for these new datasets are shown in Fig. 3d, & Fig. A2c. In brief, these data corroborate our key finding that MIA success rates are exceptionally high for a small subset of patients, while aggregate attack success does not indicate this vulnerability.

Finally, we would like to remark that there is strong evidence contradicting the referee's statement that chest radiographs cannot be used to re-identify patients. For one, *Packhäuser et al.* [7] showed that patients can be re-identified from chest radiographs with remarkably high success rates using simple AI techniques (siamese networks). Moreover, there is a general consensus in the privacy research community that the protection offered by anonymisation is generally imperfect: the re-identification of individuals is not limited to specific data modalities but is generally possible with any kind of anonymised data. *Rocher et al.* [8] recently proposed a general scaling law to model the effectiveness of re-identification techniques and showed that such techniques can scale to re-identify individuals in comparison pools comprising data from billions of individuals.

> *"- The notion of "minority" is unclear here. Both figures and descriptions lack detail on how subgroups are defined, their size, and more description about the data distribution within these subgroups in comparison to the entire dataset. This specific result - that MIAs are more successful for outliers/minority groups - has been shown in other papers, and might be expected, so more analysis is needed."*

We thank the referee for highlighting the lack of clarity and agree that the term "minority", due to its additional meaning from the social sciences, is problematic and could lead to confusion. To address this issue, we have replaced all relevant mentions of "minority" in the results section with more precise terminology (smallest group or under-represented group) to make clear that we are referring to the relative size of a group in a dataset only (as measured by the number of contributed records). Furthermore, we have modified figure captions to include information on subgroup stratification and additionally added tables summarising all available information (e.g., age, sex, disease prevalence, etc.) about the patient cohorts, as well as relevant subgroups (Tables A1 - A7). We would also like to point out that the relative size of each group is indicated in all figures showing data from subgroup comparisons (in Fig. 3 & A2 by bar colour and in Fig. A3 on the x-axis).

At this point, we would like to differentiate our work from the existing studies mentioned by the referee, which have shown that MIA success rates differ between subgroups. These studies report differences in aggregate MIA success between subgroups on small, low-dimensional datasets using shallow neural network models. Their findings thus offer limited generalizability for two reasons. First, prior work by *Carlini et al.* [9] showed that findings from MIAs against shallow neural networks trained on low-dimensional datasets generalise poorly to high-dimensional datasets and modern (deep neural network) AI models. Second, one of our key findings is that aggregate MIA success is an unsuitable measure of privacy risk, as the metric is dominated by the large number of records for which the attack only reaches

random-guessing performance. Thus, as a direct consequence of this finding, subgroup differences in aggregate attack success also offer limited insight into privacy risk differences between groups.

In contrast to the existing works mentioned by the referee, our study moves beyond subgroup differences in aggregate MIA success and investigates subgroup composition differences in the subset of records most susceptible to MIAs (99th record-level MIA AUC percentile) compared to the overall dataset. In other words, we measure how often, relatively speaking, a given subgroup appears among the records most susceptible to a MIA and compare this to the relative number of records the subgroup contributes to the overall dataset. Thus, while the conclusions of our study and the existing literature appear similar at first glance, the quantification of risk differences between groups is fundamentally different.

> *"- Is there a reason the authors did not subsequently train these models with DP and evaluate the benefit regarding MIAs? Obviously this would improve the leakage, but I am curious if they can find a sweet spot (e.g., with a weak epsilon but good MIA performance). DP is reasonably low hanging fruit for these models, and it would solidfy our understanding to know how addressable these issues are."*

We did not conduct these experiments for the original submission due to their high computational costs. However, we fully agree with the referee that investigating AI models protected by differential privacy (DP) and, more importantly, the relationship between the level of privacy protection (e.g., as measured by $\epsilon$ in $(\epsilon, \delta)$-DP) and patient-level attack success rates are of high interest to practitioners or policy makers. To directly address this concern, we conducted **extensive additional experiments** on AI models with various levels of record-level $(\epsilon, \delta)$-DP privacy protection for two datasets: EMBED (imaging) and PTB-XL (non-imaging). Data from these experiments have been integrated into the revised manuscript (see Fig. 2d & e) and suggest that, as expected, MIA success rates decrease for all patients with increasing levels of record-level DP protection. However, interestingly, we also found that the full mitigation of MIAs for all data-contributing patients requires stricter levels of privacy protection (smaller $(\epsilon, \delta)$) than previously believed [9,11]. Specifically, our results indicate that fully mitigating MIAs requires DP accounting at the patient- rather than the record-level. This suggests that record-level DP protection, which is commonly used for its simplicity across academia and industry, is not sufficient to completely prevent privacy attacks on an AI model that is trained on a dataset where one individual contributes multiple records.

> *"- The proposed extension to LR-MIA seems computationally intensive, requiring training of many models. Scalability concerns, particularly on large datasets, are not sufficiently discussed here to warrant understanding of the extensibility."*

We would like to clarify that we do not propose an extension to LR-MIA. Instead, we propose a simple and efficient technique to estimate the success rate of LR-MIAs at record/patient-level resolution. Estimating MIA success rates at patient-level resolution, by definition, requires measuring attack success rates -independently for each patient's records- across many target models (see Fig. 1c). Conducting a privacy audit at this level of granularity is thus necessarily a computationally expensive endeavour. Notably, the only other work that measures attack success at record-level granularity uses N=20,000 target models [12]. Besides the order of two magnitudes efficiency gain (20,000 vs. 200), our method has two advantages: (1) it requires

target models only, and (2) it provides standard error estimates for each patient's attack success rate. In a practical setting, an auditor can thus easily gauge the standard error of each patient's MIA success estimate and adaptively decide whether more computational resources should be allocated to reduce the estimation error further (by training more target models) or whether the current level of error is satisfactory for their use case. See our response directly below for our assessment of an extensive ablation study on computational resources and their impact on the standard error of our record-level MIA success estimates.

> *"- It is important to know the sensitivity of the score to the number of trained models required. What is a reasonable way for picking N? I see no ablations done here to confirm it, but this is important for usage in practice."*

This is an excellent question that is highly relevant to practitioners. We have conducted an extensive ablation study on $N$ (the number of target models used for record-level MIA success estimation) across all investigated datasets. Data from this ablation study (see Fig. A4) suggests that the standard error for all record-level MIA AUC scores decreases quickly with an increasing number of target models. Crucially, the standard error among the most vulnerable records (99th record-level MIA AUC percentile) decreases even faster. This effect is particularly pronounced for PTB-XL and Fitzpatrick 17k, the two datasets with the highest MIA vulnerability. This property –that the standard error drops rapidly for the 99th percentile of vulnerable records– is useful for practitioners as it allows identifying the most vulnerable records/patients with modest computational resources.

> *"- How does the proposed method perform compared to existing MIA, for instance in comparison to a calibrated LR-MIA prediction?"*

Could the referee please clarify what they mean by calibrated LR-MIA prediction? *LiRA* [9] and *RMIA* [13], the state-of-the-art MIAs we investigate in our study, are difficulty-calibrated to every target record. In essence, these attacks exploit relative changes in confidence when a record is included in/excluded from a model's training dataset. Both of these attacks (LiRA and RMIA) are constructed using a likelihood-ratio test on model confidence values and are thus likely quite close to the theoretically-optimal performance of a statistical test for membership inference (see Neyman-Pearson Lemma [14]). Since LiRA and RMIA are widely considered the most powerful existing attacks, we are unsure whether comparisons to other MIAs would yield any further insights.

> *"Minor Points*
> *-----***-----*
> *- The terms target model and shadow model appear to be used interchangeably? My understanding is that the models that are trained to replicate the target model behaviour are called shadow model. Can you clarify why you mention "training target models"?"*

We'd like to clarify this point regarding the use of "target model" and "reference model" (also referred to as "shadow model" in the wider literature). These terms have been used with great care and rigour throughout the entire manuscript to denote: models used to compute statistics needed to conduct LR-MIAs, and models used to evaluate MIA success, respectively (analogous to training and testing data in classical machine learning).

To measure MIA success, one must know the ground truth membership status, i.e., which records were actually included or excluded from the target model's training data. As is standard practice –in virtually all publications on MIAs– attack success is measured by 'training a target model' on a random subset of the training dataset, then evaluating the attack's success in aggregate across all records (as illustrated in Fig. 1b). Our study follows this established methodology but crucially measures MIA success at a finer, record/patient-level granularity. This requires evaluating attack success independently for each target record across many (N=200) target models (as shown in Fig. 1c). Note that these target models are split evenly such that for every target record, there are N=100 target models that included the record in their training dataset and N=100 target models that did not. For a more detailed explanation of our process for measuring record/patient-level MIA success, please refer to the section titled "From aggregate to patient-level privacy risk estimation."

> *"- Citation error: Page 4, first line contains an incorrect citation for LR-MIA."*

Thank you for spotting this error; we have corrected it in the revised manuscript.

> *"- The paper shows that outliers are more vulnerable to MIA, but it's unclear how inference models treat outlier samples not present in the training set. I'm curious to know if such samples are easier to identify or are systematically given higher probability of being part of the training data?"*

The referee's concern that there is a lack of clarity around how LR-MIAs treat outlier samples that were not part of the training dataset is unfounded, as this point has been addressed in prior work by *Carlini et al.* [9]. The key innovation behind LR-MIAs, and the reason why this type of attack is so powerful, is that the attack is independently calibrated for each and every target record. In essence, LR-MIAs compare, for each target record, the relative change in confidence when the record is included in/excluded from a model's training dataset. This means that outliers, which were not part of the training dataset, are not assigned a higher probability of being part of the training data than any other 'normal' record. On the other hand, a large change in confidence is expected for outliers that were part of the training dataset; thus, naturally explaining why MIAs on such records are so effective. While it is true that earlier MIAs (such as, e.g., the loss threshold attack by *Yeom et al.* [15]) had a tendency for the behaviour on outlier records described by the referee, LR-MIAs do not suffer from this problem.

> *"- A similar question arises for minority groups—is the inference model simply worse at predicting membership for them due to systematic model bias?"*

We do not understand this question and would kindly ask the referee to clarify what exactly they mean.

> *"- The discussion says "efforts to collect diverse training datasets, which are urgently needed to build unbiased AI models which offer equitable performance on diverse patient populations, will require improving data- sharing trust relationships with minority groups" While this can be very true, I didn't see anywhere in the paper showing the impact of bias in the data with inference attack. If there is such finding, it would be very interesting to highlight it."*

While we share the referee's opinion that this is an interesting topic to investigate, the assessment of AI performance bias hinges on choosing a fairness definition that is appropriate and relevant to the specific clinical setting in which a model is deployed. Picking a relevant fairness definition is thus application-specific and would likely yield different technical definitions/metrics for each of the datasets investigated in our study. As a result, we deem this request beyond the scope of the paper and have removed the relevant sentence from the discussion section to improve its clarity and avoid confusion.

## Referee #2 (Remarks to the Author):

> *"This manuscript presents a systematic study of disparate privacy risks in medical AI, focusing on how membership inference attacks (MIAs) affect demographic subgroups differently. Using a variety of clinical datasets (including MIMIC-IV and MedMNIST) and machine learning models (neural networks, LightGBM, logistic regression), the authors find that individuals from underrepresented or marginalized demographic groups (e.g., racial minorities, the elderly, and people with rare health conditions) often face higher privacy risks in AI models. Importantly, these disparities persist even when group-level membership inference risks appear similar, highlighting a previously underappreciated layer of privacy inequity in clinical ML.*
>
> *Originality and Significance:*
> *The work is original and highly significant. While membership inference attacks have been studied in general contexts, the exploration of demographic disparities in privacy vulnerability is novel and timely. The paper offers a critical perspective on AI fairness by linking data protection and equity concerns. It extends and complements recent literature on fairness and privacy in ML, but focuses uniquely on subgroup-differentiated privacy vulnerabilities in healthcare, which has not been comprehensively explored. The findings are consequential for policy and practice, especially in regulated domains like healthcare where both fairness and privacy are essential.*
>
> *Data and Methodology:*
> *The study uses publicly available, well-established datasets (MIMIC-IV, MedMNIST, UTHealth Claims) with sufficient breadth and clinical realism to support generalizable claims. The methodology is robust: the use of multiple models, both black-box and white-box MIAs, and extensive subgroup analyses lend credibility to the results. The authors clearly define the statistical treatment of subgroup disparities (e.g., per-individual vs. aggregate privacy risk) and make a compelling case for disaggregating privacy risk evaluations."*

The above sections marked in yellow are factually incorrect statements about the datasets/models/methods used in our original submission; none of these datasets or techniques were used in our original submission.

> *"The presentation of the methods is generally strong, though some details (e.g., model hyperparameters, training epochs, and specific attack model structures) could be clarified in an appendix or supplementary material for reproducibility."*

All methodological details that the referee requests here are described in sufficient detail to reproduce our findings and can be found in the Methods section (see section “General model training details” & Table 1). We would kindly ask the referee to point out exactly which additional details are missing, and we will add them.

> *“Statistical Considerations:*
> *The statistical approach is appropriate for the questions posed. The authors correctly distinguish between individual-level and aggregate-level privacy risks, and quantify disparities using both absolute and relative comparisons. Uncertainty is reasonably addressed using confidence intervals and significance testing for key results. However, more formal statistical testing for between-group differences (e.g., bootstrapped CIs or adjusted p-values for multiple comparisons across subgroups) would further strengthen claims of disparity.”*

We thank the referee for the suggested improvement of our methodology in order to ensure reliable statistical conclusions. To directly address this comment, we have corrected for multiple comparisons across all subgroup comparisons performed for a single dataset using the Bonferroni method (see Fig. 3 & A2 for the updated results).

> *“Conclusions:*
> *The conclusions are supported by the data and analyses. The key claims—that (1) membership inference risks are not evenly distributed across demographic groups and (2) underrepresented groups tend to bear greater risks—are robust across datasets, models, and attack types. The implications for healthcare AI governance, particularly regarding data sharing, regulatory oversight, and ethical model deployment, are well grounded. Nonetheless, some claims (e.g., regarding the ethical obligation to assess subgroup privacy risk) could be more explicitly supported by normative frameworks or legal precedent (e.g., GDPR’s fairness and data protection principles).”*

We fully agree with the referee on the importance of the ethical and legal implications of our findings. Our expertise, however, is concentrated on machine learning for medicine, and we've thus consciously refrained from commenting on the legislative implications of our work, such as for the GDPR. We believe it's crucial for legal experts to lead the interpretation of our findings in this domain. That said, we would be happy to provide explanations of the technical details to legal experts or policymakers to help guide the interpretation of our findings.

> *“Suggested Improvements:*
> *- Consider evaluating mitigation techniques (e.g., differential privacy, adversarial training) across subgroups to explore whether disparities persist even when using common defenses.*
> *- Include subgroup sizes and baseline outcome distributions in each dataset to contextualize disparity results.*
> *- Include an appendix with detailed hyperparameters and training configurations for reproducibility.*
> *- Strengthen the conclusion with more direct recommendations for regulators, IRBs, and clinical ML practitioners.”*
>
> *… (remaining positive comments omitted for brevity)...*

We have integrated all of the above-mentioned suggestions into the revised manuscript. Please see the following sections:

- Investigation of models protected by differential privacy (Fig. 2d & e)
- Subgroup sizes and detailed information about patient cohorts (see Table A1-A7)
- Detailed hyperparameters and training configurations (Table 1)
- More concrete recommendations for models protected by differential privacy (discussion section)

The relevant, updated section in the discussion now reads:

*“Our experimental data confirmed that stronger levels of DP protection effectively reduce MIA success for all data-contributing patients. However, interestingly, we also observed that mitigating MIAs requires stronger levels of DP protection than previously thought [9,11]. Specifically, our results indicate that fully mitigating MIAs for all patients requires implementing DP protection at the patient- rather than the record-level.“*

## Additional General Remark:

While this was not requested by any of the referees, we have made a minor technical improvement in the estimation procedure for patient-level MIA success. Our method requires training AI models on randomly sampled subsets of the training dataset. Before the modification, these subsets were sampled without taking into account which patient the included/excluded records belonged to. This led to an underestimation of MIA success for patients who contribute many similar records. Our improved approach now samples subsets at the patient-level, thus ensuring that either all, or none, of a patient's records are included to train a single model.

We are confident that this change from record- to patient-level sampling substantially improves the estimation of attack success for patients who contribute many similar records. Please note that this change has caused slight deviations in some of the experimental data compared to the original submission. All major trends and findings, however, remain consistent.

## Addressing the DURC Concerns:

To begin, we would like to point out that our study does not introduce any novel methodology that could be used to cause privacy harm to individuals. The MIAs we investigate were proposed in [9, 13] and have been available as open-source software for several years now. Instead, our study proposes a method to granularly measure the success rates of this existing type of attack at patient-level resolution. This allows auditors to identify the most vulnerable records/patients in a dataset, a prerequisite for building AI systems that offer meaningful protection against privacy attacks.

Next, we would like to address the potential negative impacts that could arise from the widespread dissemination of knowledge that MIAs against AI models are exceptionally effective for individual records/data contributors. This could lead to an increase in the number of attack attempts against medical AI models, which, if successful, can lead to the disclosure of sensitive patient information. While we acknowledge that this is a serious concern (particularly for large, generative models), we strongly believe that withholding knowledge of this vulnerability would have a much greater negative impact. Concurrent research by *Aerni et al.* [12] has presented similar record-level MIA success results to ours, albeit on natural image datasets, and thus it would only be a matter of time before other researchers arrive at the same conclusions as those presented in our work.

Overall, our work provides a positive outlook as we also demonstrate how the discovered privacy vulnerabilities can be mitigated. With the revised manuscript, we now present empirical evidence that increasing levels of record-level DP protection effectively reduces MIA success for all data-contributing patients. Crucially, however, our results also indicate that fully mitigating MIAs for all data-contributing patients requires DP accounting at the patient- rather than the record-level. This information could be valuable to policymakers as it indicates that record-level DP accounting, which is commonly used across academia and industry for its simplicity, is not sufficient to fully prevent privacy attacks against AI models trained on datasets where individual patients contribute multiple records.

Given the above points, we strongly believe that the benefit of making the results of our research publicly available outweighs any potential risks.

## References:


[1] Yoo, Seong-Keun, et al. "Prediction of checkpoint inhibitor immunotherapy efficacy for cancer using routine blood tests and clinical data." *Nature Medicine* 31.3 (2025): 869-880.

[2] Carlini, Nicolas, et al. "Extracting training data from diffusion models." *32nd USENIX Security Symposium (USENIX Security 23)*. 2023.

[3] Carlini, Nicholas, et al. "Extracting training data from large language models." *30th USENIX Security Symposium (USENIX Security 21)*. 2021.

[4] Nasr, Milad, et al. "Scalable extraction of training data from aligned, production language models." *The Thirteenth International Conference on Learning Representations*. 2025.

[5] Wagner, Patrick, et al. "PTB-XL, a large publicly available electrocardiography dataset." *Scientific data* 7.1 (2020): 1-15.

[6] Xie, Feng, et al. "Benchmarking emergency department prediction models with machine learning and public electronic health records." *Scientific Data* 9.1 (2022): 658.

[7] Packhäuser, Kai, et al. "Deep learning-based patient re-identification is able to exploit the biometric nature of medical chest X-ray data." *Scientific Reports* 12.1 (2022): 14851.

[8] Rocher, Luc, Julien M. Hendrickx, and Yves-Alexandre de Montjoye. "A scaling law to model the effectiveness of identification techniques." *Nature Communications* 16.1 (2025): 347.

[9] Carlini, Nicholas, et al. "Membership inference attacks from first principles." *2022 IEEE symposium on security and privacy (SP)*. IEEE, 2022.

[10] Fawcett, Tom. "An introduction to ROC analysis." *Pattern recognition letters* 27.8 (2006): 861-874.

[11] Nasr, Milad, et al. "Adversary instantiation: Lower bounds for differentially private machine learning." *2021 IEEE Symposium on security and privacy (SP)*. IEEE, 2021.

[12] Aerni, Michael, Jie Zhang, and Florian Tramèr. "Evaluations of machine learning privacy defenses are misleading." *Proceedings of the 2024 on ACM SIGSAC Conference on Computer and Communications Security*. 2024.

[13] Zarifzadeh, Sajjad, Philippe Liu, and Reza Shokri. "Low-Cost High-Power Membership Inference Attacks." *International Conference on Machine Learning*. PMLR, 2024.

[14] Neyman, Jerzy, and Egon Sharpe Pearson. "On the problem of the most efficient tests of statistical hypotheses." *Philosophical Transactions of the Royal Society of London. Series A, Containing Papers of a Mathematical or Physical Character* 231.694-706 (1933): 289-337.

[15] Yeom, Samuel, et al. "Privacy risk in machine learning: Analyzing the connection to overfitting." *2018 IEEE 31st computer security foundations symposium (CSF)*. IEEE, 2018.

[16] World Health Organization (WHO), “World report on social determinants of health equity” *WHO, 2025*

[17] Seyyed-Kalantari, Laleh, et al. "Underdiagnosis bias of artificial intelligence algorithms applied to chest radiographs in under-served patient populations." *Nature Medicine* 27.12 (2021): 2176-2182.

[18] Daneshjou, Roxana, et al. "Disparities in dermatology AI performance on a diverse, curated clinical image set." *Science Advances* 8.31 (2022): eabq6147.

[19] Obermeyer, Ziad, et al. "Dissecting racial bias in an algorithm used to manage the health of populations." *Science* 366.6464 (2019): 447-453.

# Response to referees: 2025-01-00537

We would like to thank the referees again for taking the time to provide such detailed and helpful feedback on our manuscript. Please find our point-by-point response to their comments below.

**Referee #1:**

> *"One of my major remaining concerns, which was unaddressed in the response, is that several strands of prior work already document that membership-inference risk is heterogeneous. For example, disparate vulnerability across subpopulations has been analyzed with statistical tests and mitigation studies (e.g., Disparate Vulnerability to Membership Inference Attacks, https://arxiv.org/pdf/1906.00389); class-dependent variability in attack success has been measured at the label level (https://ieeexplore.ieee.org/document/9230385 ); and individual-level susceptibility was observed in early MIA formulations ( https://arxiv.org/pdf/1610.05820). For a paper of this magnitude, it is crucial that you make explicit how your LR-MIA setup, metrics, and clinical use case relate to and extend these lines, and highlight where your choices align with or differ from prior findings."*

We agree with the referee that contextualising our findings within the related literature on MIA risk heterogeneity is essential to the completeness of our paper. To this end, we have added an additional paragraph directly at the beginning of the Discussion section in our revised manuscript. We have clarified three key aspects of our findings that advance the field beyond early reports of MIA risk heterogeneity:

1. **Unit of analysis (patient vs. record):** While prior works [33, 34] documented risk heterogeneity at the single-record level, clinical reality requires a patient-level view. We provide extensive patient-level MIA success data across diverse real-world clinical datasets, marking the first time such data have been presented.
2. **Metric suitability (extreme vs. aggregate):** Our data show that aggregate MIA success is an unsuitable measure of privacy risk, casting doubt on subgroup analyses [35, 36] that relied on aggregate success rates. Our work demonstrates that extreme risk (99th percentile) is a more informative metric for privacy audits.
3. **Real-world data and scale:** We confirm that MIA vulnerabilities previously observed on low-dimensional benchmark datasets hold true and are arguably more critical in large clinical datasets. In contrast to prior MIA research, the datasets we investigate are representative of those used to train medical AI models deployed to the real world.

The corresponding paragraph in the Discussion section now reads as follows:

*"We present data from the first patient-level privacy audit of diagnostic medical AI models. Our findings confirm early observations of MIA risk heterogeneity [33-36] and, at the same time, substantially advance prior AI privacy auditing efforts along three key dimensions. First, our work marks a shift towards patient-level risk assessment, which is crucial for real-world clinical datasets where individuals often contribute multiple, similar records. Second, we demonstrate that aggregate success rates, as used in the standard evaluation protocol and previous subgroup analyses [35,36], underestimate true privacy risks. Third, we confirm that MIA vulnerabilities previously observed on low-dimensional benchmark datasets [2-4,33-36] are present, and arguably more critical, in large representative clinical datasets. Below, we briefly discuss our findings and their implications in more detail."*

[2] Shokri, R., Stronati, M., Song, C., Shmatikov, V.: Membership inference attacks against machine learning models. 2017 IEEE Symposium on Security and Privacy (SP) (2017).

[3] Carlini, N., Chien, S., Nasr, M., Song, S., Terzis, A., Tramer, F.: Membership inference attacks from first principles. 2022 IEEE Symposium on Security and Privacy (SP) (2022). IEEE

[4] Zarifzadeh, S., Liu, P., Shokri, R.: Low-cost high-power membership inference attacks. Proceedings of the 41st International Conference on Machine Learning (2024)

[33] Long, Y., Wang, L., Bu, D., Bindschaedler, V., Wang, X., Tang, H., Gunter, C.A., Chen, K.: A pragmatic approach to membership inferences on machine learning models. IEEE European Symposium on Security and Privacy (EuroS&P) (2020)

[34] Aerni, M., Zhang, J., Tramer, F.: Evaluations of machine learning privacy defenses are misleading. Proceedings of the 2024 on ACM SIGSAC Conference on Computer and Communications Security (2024)

[35] Kulynych, B., Yaghini, M., Cherubin, G., Veale, M., Troncoso, C.: Disparate vulnerability to membership inference attacks. Proceedings on Privacy Enhancing Technologies (2019)

[36] Chang, H., Shokri, R.: On the privacy risks of algorithmic fairness. IEEE European Symposium on Security and Privacy (EuroS&P) (2021)

> *“Below are the follow-up questions I have in response to the updated work, noted per comment:*
> *--------------*
> *Original comment: The link between MIA success and actual privacy risk is not clearly established. If the adversary already has access to the data, what additional risk does membership inference pose? Again, see "Can LLMs Keep a Secret? Testing Privacy Implications of Language Models via Contextual Integrity Theory" for an examination of privacy in context. Specifically, is the issue that models reveal private information, or that models reveal private information in contexts that humans would not?*
>
> *Updated: The immunotherapy example is constructed narrowly so as to be strong and make your point well; it also highlights that privacy risk is nuanced and depends on many factors, for instance the population the model was trained on and related dataset/model characteristics. The text should clarify this nuance explicitly in the Introduction and revisit it in the Discussion so the manuscript accurately reflects how MI success rates translate into patient privacy risk across different settings, rather than raising a uniform concern.“*

We agree with the referee that it is crucial to clearly communicate the context-specific nature of MIA privacy risks to prevent the misinterpretation of our results. To this end, we have now added the following paragraph to the introduction:

*“Privacy attacks on an AI model can enable detailed inferences about the individuals who contributed to its training data. For example, a membership inference attack (MIA) [2] attempts to determine whether a specific patient's data was included in a model's training dataset. The extent to which this constitutes a privacy violation is nuanced and depends on factors such as the underlying training population and the model's deployment context. While*

*inferring membership for a model trained on a general population may be benign, doing so for a model trained on a narrow, disease-specific cohort acts as a direct proxy for sensitive medical information. For example, a successful MIA against the model by Yoo et al. [10], which predicts anti-cancer immunotherapy efficacy from routine blood test data, reveals that an individual has cancer. Notably, in addition to their direct use against diagnostic AI models, MIAs are also used as a core building block in data-extraction attacks against generative models [5-7]. Overall, MIA vulnerability thus serves as a key indicator for generative AI privacy risk."*

We revisit this point in the conclusion with clear recommendations for practitioners:

*"In summary, we present evidence that MIAs are exceptionally effective at compromising the privacy of individual data-contributing patients. Given this vulnerability, medical AI models and their deployment contexts should be assessed for the sensitive information that attackers could obtain by successfully inferring training dataset membership. To prevent privacy harm, we recommend that vulnerable models be protected by verifiable risk mitigation strategies and/or strict access controls."*

In addition to these two sections explicitly aimed at communicating the nuances of MIA privacy risks, we have replaced all general terminology (e.g., AI privacy risks) in the results and discussion section with more specific terminology (e.g., MIA risk/success). This should further help readers gain a clear, accurate understanding of our findings while keeping the manuscript accessible to a wide audience.

[2] Shokri, R., Stronati, M., Song, C., Shmatikov, V.: Membership inference attacks against machine learning models. 2017 IEEE Symposium on Security and Privacy (SP) (2017).

[5] Carlini, N., Tramer, F., Wallace, E., Jagielski, M., Herbert-Voss, A., Lee, K., Roberts, A., Brown, T., Song, D., Erlingsson, U., et al.: Extracting training data from large language models. 30th USENIX Security Symposium (USENIX Security 21) (2021)

[6] Carlini, N., Hayes, J., Nasr, M., Jagielski, M., Sehwag, V., Tramer, F., Balle, B., Ippolito, D., Wallace, E.: Extracting training data from diffusion models. 32nd USENIX Security Symposium (USENIX Security 23) (2023)

[7] Nasr, M., Rando, J., Carlini, N., Hayase, J., Jagielski, M., Cooper, A.F., Ippolito, D., Choquette-Choo, C.A., Tramer, F., Lee, K.: Scalable extraction of training data from aligned, production language models. The Thirteenth International Conference on Learning Representations (2025)

[10] Yoo, S.-K., Fitzgerald, C.W., Cho, B.A., Fitzgerald, B.G., Han, C., Koh, E.S., Pandey, A., Sfreddo, H., Crowley, F., Korostin, M.R., et al.: Prediction of check-point inhibitor immunotherapy efficacy for cancer using routine blood tests and clinical data. Nature Medicine (2025)

*Original comment: The paper is focusing on MIA for medical imaging models only - which lacks a clear argument for harm, e.g., in leaking someone's chest x-ray because that cannot be used to reconstruct identity or otherwise identify someone. For example, a head MRI example is a different case where we can indeed reconstruct from chest x-rays. Even then I think MIAs are a privacy concern that needs to be justified for healthcare models vs. reconstruction attacks. The more concerning result would be if it held for EHR models.*

*Updated: The new results on MIMIC and PTB-XL could substantially strengthen the paper, but require clarity on how the EHR attacks were conducted. Specifically, do success rates reflect attacks using the full patient record or partial records/subsets of features and time windows? This distinction matters enormously for interpreting privacy risk: successful membership inference given complete records implies a different (extremely low) real-world risk than success when only partial records are available.*

This is an excellent point. The extent to which MIAs are possible with partial target records is crucial for assessing the magnitude of risk that MIAs pose to EHR or ECG models in the real world.

For the MIMIC-IV-ED dataset, we follow the established data pre-processing methodology of *Xie et. al.* [59]. This means that EHR models are trained to predict hospitalisation outcomes based on 64 clinical features (see Table on page 7 for details). These features comprise patient history data, as well as core information on vital signs, comorbidities, chief complaints and other relevant information at triage and at ED discharge. As is the case for all investigated datasets, the MIA success data we report assume an attacker with full access to target records, i.e., all 64 clinical features in the case of MIMIC-IV-ED. For EHR and ECG models, we fully agree with the referee that MIAs with complete target records entail lower real-world risk than those with only partial records.

To provide quantitative evidence on the extent to which MIAs remain possible under partial target record access, we have conducted additional experiments for MIMIC-IV-ED and PTB-XL. Results from these experiments suggest that MIA success under partial data access decreases only slightly and remains exceptionally high for some patients. Remarkably, in the MIMIC-IV-ED dataset, we find that some patients still experience high MIA risk even when an attacker has access to only 20 of the 64 clinical features, comprising only a patient's triage vital signs, age, sex, and chief complaints. Similarly, for PTB-XL (12-lead ECG data), we find that some patients remain vulnerable to MIAs even when an attacker has access only to the signal from Lead I. Given that modern smart watches aim to generate data that is similar to Lead I signals, this suggests it may be possible to conduct a MIA against a model trained on clinical 12-lead ECG data using only data collected by a patient's smart watch. The data from these new experiments have been integrated into the revised manuscript (see Extended Data Fig. 5). The relevant section in the updated results section now reads as follows:

*"For the two non-imaging datasets, MIMIC-IV-ED (electronic health records) and PTB-XL (electrocardiograms), we simulate attack settings in which an attacker has only partial access to the target record (Extended Data Fig. 5). Generally, we find that MIA success*

*under partial data access decreases only slightly and remains exceptionally high for some patients. Notably, MIAs remain remarkably effective in realistic attack settings where an attacker has extremely limited information about the target record, such as, e.g., only a patient's age, sex, chief complaints and vital signs (MIMIC-IV-ED), or only lead I signal from a patient's twelve-lead electrocardiogram (PTB-XL).”*

For details on the experimental setup, we refer to the revised manuscript's Methods section, “Measuring MIA success with partial target records.”

[59] Xie, F., Zhou, J., Lee, J.W., Tan, M., Li, S., Rajnthern, L.S., Chee, M.L., Chakraborty, B., Wong, A.-K.I., Dagan, A., et al.: Benchmarking emergency department prediction models with machine learning and public electronic health records. Scientific Data (2022)

REDACTED

*Screenshot from Xie et. al [59], Scientific Data,* Extended Data Table 1*.*

*"Original comment: How does the proposed method perform compared to existing MIA, for instance in comparison to a calibrated LR-MIA prediction?*

*Updated: I would like to clarify the original question. By this question I meant taking the per-record LR (LiRA/RMIA) scores (optionally calibrated to membership probabilities, but this is not strictly required) and then examining the extreme tail of those scores to see who is most at risk. Could this potentially characterize those extremes to be latent subpopulations? This matters because, for instance, a patient with a very atypical diagnosis may not fall into any predefined subgroup yet still sit in the extreme-risk tail; clustering or describing features of the tail (e.g., diagnosis codes, test panels, site/device attributes) would reveal such cases."*

Thank you for clarifying your initial question; this is an interesting idea. We would like to note that we already report extensive data on composition differences between the extreme MIA risk tail and the general training population across all investigated datasets and most available metadata (Fig. 3 and Extended Data Fig. 2). Thus, we already provide the data the referee requests here, albeit indirectly. To address the referee's comment and improve the reproducibility of our results, we have added tables that directly describe features of the tail, i.e., summary statistics on the extreme MIA risk tail for each of the investigated datasets (see Supplementary Data Tables 8-14). In essence, this is the raw data underlying the Chi-Squared test results reported in Fig. 3 and Extended Data Fig. 2. Attempts to cluster data from the extreme MIA risk tail, as the referee suggested, did not yield any insights that held consistently across datasets.

At this point, we would like to note that multiple examples in Fig. 3 and Extended Data Fig. 3 suggest that latent subpopulations exist within the extreme MIA risk tail. Consider, for example, data for EMBED shown in Fig. 3c. Models for EMBED are trained to predict breast density given a mammogram, and despite these models never having direct access to tumour findings, mammograms with benign (BI-RADS 2) or potentially cancerous (BI-RADS 4) tumour findings are significantly over-represented in the extreme MIA risk tail compared to the overall dataset (+60% and +1,179% relative change to the overall dataset, respectively). Similar examples that provide further support for the existence of latent subgroups in the MIA risk tail are found across most of the investigated datasets. We have added additional text to the results section to highlight these findings:

*"Consider, for example, EMBED, a mammography dataset comprising mostly negative findings, i.e., unremarkable mammograms of healthy breasts with no indication of a tumour. Models for this dataset are trained to predict breast density, and thus never have direct access to tumour findings. Despite this, benign tumour findings (BI-RADS-2) and tumour findings suspicious of malignancy (BI-RADS-4) account for a disproportionately large share of the most vulnerable records (+60% and +1,179% relative change to the overall dataset, respectively). Similarly, otherwise relatively uncommon images of almost entirely fatty (BI-RADS-A) or extremely dense (BI-RADS-D) breasts also occur disproportionately frequently (+90% and +755%, respectively)."*

These findings are discussed further in the discussion section:

*“We found significant differences in the frequency with which patients from different subgroups experience extreme MIA risk. The fact that some of these groups (e.g., self-reported race subgroups in chest radiographs) are not readily distinguishable by human experts raises concerns that MIA risk differences, which likely exist beyond the stratification variables we investigated, may pass unnoticed in practice.”*

Finally, we would like to remark that verifying latent subpopulations beyond the available metadata we already report would require extensive manual labelling by medical experts, which we deem out of scope. To enable other researchers to reproduce our findings and, further, conduct their own investigations, we will make the anonymous patient identifiers for patients in the extreme MIA risk tails publicly available in our GitHub and Zenodo repositories. Publishing these anonymous patient identifiers will allow any researcher with access to the respective datasets to conduct their own investigations into factors driving MIA tail risk. Crucially, this avoids directly sharing underlying patient data and thus conforms with the data access agreements for five out of the seven investigated datasets. For CheXpert and EMBED, the two datasets where ambiguity regarding this point exists in the data access agreements, we have contacted the relevant parties to obtain written clarification on whether we may publish a subset of anonymous patient identifiers.

> *Original comment: The paper shows that outliers are more vulnerable to MIA, but it’s unclear how inference models treat outlier samples not present in the training set….. A similar question arises for minority groups.Is the inference model simply worse at predicting membership for them due to systematic model bias?*
>
> *Updated: Regarding my concern about attack performance and its relation to data distribution, I’m clarifying my question. For example, a common driver of subgroup vulnerability is overfitting - where minority groups (with fewer samples or distinctive features) are often easier for the model to memorize. While you note efforts to prevent overfitting (“we undertake every effort…countermeasures to prevent overfitting”), the analysis doesn’t disentangle how much of the observed MIA success reflects intrinsic vulnerability versus data effects like overfitting. To clarify the root cause, you can for example report subgroup-wise generalization metrics (test accuracy/loss), and member vs non-member loss distributions, and compare them to overall performance. Overall, brief correlation analysis between subgroup test error (or calibration) and LR-MIA power would make the causal story more convincing.*

We thank the referee for the clarification and note that, while we also see this as an interesting research question, we regard it as tangential to the findings presented in our paper.

For one, there is substantial evidence from prior research suggesting that memorisation and overfitting can and do occur as distinct phenomena in deep neural networks [38,39]. Concretely, *Feldman and Zhang* [38,39] show that memorising records from a rare subgroup improves generalisation performance for that same subgroup at test time. Thus, it is likely that a correlation analysis between subgroup generalisation performance and MIA risk could

be misleading. These findings from prior work provide direct counter-evidence to the referee's claim of a causal link between subgroup generalisation performance and MIA risk.

In addition to these conceptual limitations, practical limitations prevent us from performing the requested analysis reliably. Our study adheres to official dataset splits wherever possible to ensure comparability with existing literature. Consequently, the resulting test partitions are often too small to yield statistically reliable estimates of subgroup-specific error rates or, in some cases, lack the necessary metadata for stratification altogether. To address this, we would need to restructure the dataset splits to increase the test set sizes. However, this would not only necessitate retraining all target models, incurring substantial computational cost, but could also introduce methodological issues. As our target models are trained on random 50% subsets of the data, increasing the test set size would necessarily decrease the available training data. This could render the target models non-representative of state-of-the-art performance, thus ultimately undermining the validity of the attack evaluation.

[38] Feldman, V.: Does learning require memorization? A short tale about a long tail. Proceedings of the 52nd Annual ACM SIGACT Symposium on Theory of Computing (2020)

[39] Feldman, V., Zhang, C.: What neural networks memorize and why: Discovering the long tail via influence estimation. Advances in Neural Information Processing Systems (2020)

> *“Original comment: The discussion says “efforts to collect diverse training datasets, which are urgently needed to build unbiased AI models which offer equitable performance on diverse patient populations, will require improving data- sharing trust relationships with minority groups” While this can be very true, I didn’t see anywhere in the paper showing the impact of bias in the data with inference attack. If there is such finding, it would be very interesting to highlight it.*
>
> *Updated: I agree that in practice the assessment of AI performance bias depends on choosing a fairness definition that is appropriate and relevant to the specific clinical setting, because this is application-specific. However, the authors can simply choose a plausible single - or set of - applications and demonstrate the impact of bias with inference attack in that setting? If the authors would rather remove all mentions of ”*

Please see our response on the previous page for an explanation of the conceptual and practical limitations preventing us from conducting the requested analysis faithfully. We have clarified the discussion section to prevent the misinterpretation of our presented results, which do not include data on diagnostic performance differences between subgroups. Statements about the implications of our findings for minority groups' willingness to contribute training data are phrased appropriately speculatively.

# Response to referees: 2025-01-00537

We would like to thank the referees again for taking the time to provide such detailed and helpful feedback on our manuscript. Please find our point-by-point response to their comments below.

**Referee #1:**

> *"I have no further major concerns, but I still think the tone is overly aggressive about risks without some context for practical risk (see prior reviews). I would appreciate this being addressed in the final version."*

We have made extensive efforts to clarify our findings and to tone down the language's aggressiveness throughout the manuscript. Most notably, we have modified the tone of the abstract and the introduction. Besides toning down the language in the abstract, we have added two short additional sentences to clarify our findings and provide the necessary context for their correct interpretation. We have also toned down and clarified the last sentence in the abstract, which now offers a more cautious outlook on how our findings could affect future AI privacy risk assessments.

In light of the referee's comment, we have also removed all text on the implications of our findings for generative AI privacy risk assessments from the introduction. Since we do not present any data on MIA success against generative models (our data only spans diagnostic AI models), we felt that leaving the implications of our findings to generative AI models to the discussion section would further help avoid the misinterpretation of our results.
